# Polymorphisms Near *TBX5* and *GDF7* Are Associated With Increased Risk for Barrett’s Esophagus

**DOI:** 10.1053/j.gastro.2014.10.041

**Published:** 2015-02

**Authors:** Claire Palles, Laura Chegwidden, Xinzhong Li, John M. Findlay, Garry Farnham, Francesc Castro Giner, Maikel P. Peppelenbosch, Michal Kovac, Claire L. Adams, Hans Prenen, Sarah Briggs, Rebecca Harrison, Scott Sanders, David MacDonald, Chris Haigh, Art Tucker, Sharon Love, Manoj Nanji, John deCaestecker, David Ferry, Barrie Rathbone, Julie Hapeshi, Hugh Barr, Paul Moayyedi, Peter Watson, Barbara Zietek, Neera Maroo, Laura Gay, Tim Underwood, Lisa Boulter, Hugh McMurtry, David Monk, Praful Patel, Krish Ragunath, David Al Dulaimi, Iain Murray, Konrad Koss, Andrew Veitch, Nigel Trudgill, Chuka Nwokolo, Bjorn Rembacken, Paul Atherfold, Elaine Green, Yeng Ang, Ernst J. Kuipers, Wu Chow, Stuart Paterson, Sudarshan Kadri, Ian Beales, Charles Grimley, Paul Mullins, Conrad Beckett, Mark Farrant, Andrew Dixon, Sean Kelly, Matthew Johnson, Shahjehan Wajed, Anjan Dhar, Elinor Sawyer, Rebecca Roylance, Lynn Onstad, Marilie D. Gammon, Douglas A. Corley, Nicholas J. Shaheen, Nigel C. Bird, Laura J. Hardie, Brian J. Reid, Weimin Ye, Geoffrey Liu, Yvonne Romero, Leslie Bernstein, Anna H. Wu, Alan G. Casson, Rebecca Fitzgerald, David C. Whiteman, Harvey A. Risch, David M. Levine, Tom L. Vaughan, Auke P. Verhaar, Jan van den Brande, Eelke L. Toxopeus, Manon C. Spaander, Bas P.L. Wijnhoven, Luc J.W. van der Laan, Kausilia Krishnadath, Cisca Wijmenga, Gosia Trynka, Ross McManus, John V. Reynolds, Jacintha O’Sullivan, Padraic MacMathuna, Sarah A. McGarrigle, Dermot Kelleher, Severine Vermeire, Isabelle Cleynen, Raf Bisschops, Ian Tomlinson, Janusz Jankowski

**Affiliations:** 1 Wellcome Trust Centre for Human Genetics, University of Oxford, Oxford, UK; 2 Plymouth University Peninsula School of Medicine and Dentistry, Plymouth, Devon, UK; 3 Centre of Biostatistics, Bioinformatics and Biomarkers, Plymouth University Peninsula Schools of Medicine and Dentistry, Plymouth, Devon, UK; 4 Department of Gastroenterology and Hepatology, Erasmus MC, University Medical Centre Rotterdam, Rotterdam, The Netherlands; 5 Department of Digestive Oncology, University Hospital Gasthuisberg, Leuven, Belgium; 6 Department of Pathology, Leicester Royal Infirmary, Leicester, UK; 7 Department of Cellular Pathology, Warwick Hospital, Warwick, UK; 8 Department of Oral Biological and Medical Sciences, University of British Columbia, Vancouver, British Columbia, Canada; 9 Department of Gastroenterology, Wansbeck General Hospital, Ashington, Northumberland, UK; 10 William Harvey Research Institute, The Ernest Cooke Vascular & Microvascular Unit, Centre for Clinical Pharmacology, St Bartholomew's Hospital, London, UK; 11 Centre for Statistics in Medicine and Oxford Clinical Trials Research Unit, Oxford, UK; 12 Centre for Digestive Diseases, Queen Mary University of London, London, UK; 13 Department of Gastroenterology, Leicester General Hospital, Leicester, UK; 14 Department of Oncology, New Cross Hospital, Royal Wolverhampton Hospitals NHS Trust, Wolverhampton, UK; 15 Department for Gastroenterology, Leicester Royal Infirmary, Leicester, UK; 16 Gloucestershire Royal Hospital, Great Western Road, Gloucester, UK; 17 Department of Upper GI Surgery, Gloucestershire, Royal Hospital, Gloucester, UK; 18 Department of Medicine, McMaster HC, Hamilton Ontario, Canada; 19 School of Medicine, Dentistry, and Biomedical Sciences, Centre for Public Health, Queens University Belfast, NI; 20 University of Southampton, Southampton General Hospital, Tremona Road, Southampton, UK; 21 Lancashire Teaching Hospitals NHS Foundation Trust, Royal Preston Hospital, Lancashire, UK; 22 General Surgery, Countess of Chester Hospital, Chester, UK; 23 Southampton University Hospitals NHS Trust, Southampton, UK; 24 Wolfson Digestive Diseases Centre, Queens Medical Centre, Nottingham, UK; 25 Worcestershire Acute Hospitals NHS Trust, Alexandra Hospital, Redditch, UK; 26 Department of Gastroenterology, Royal Cornwall Hospital, Truro, Cornwall, UK; 27 Macclesfield General Hospital, Macclefield, Cheshire, UK; 28 Department of Gastroenterology, Sandwell General Hospital, Lyndon, West Bromwich, UK; 29 Department of Gastroenterology, University Hospital of Coventry, Coventry, UK; 30 Department of Gastroenterology, Leeds General Infirmary, Leeds, UK; 31 Department of Clinical Pharmacology University of Oxford, Oxford, UK; 32 School of Biomedical & Healthcare Sciences, Plymouth University Peninsula Schools of Medicine and Dentistry, Plymouth, UK; 33 Gastroenterology, Royal Albert Edward Infirmary NHS Trust, Wigan, UK; 34 GI Science Centre, Salford Royal NHS Foundation Trust, University of Manchester, Salford, UK; 35 Department of Gastroenterology and Hepatology, Department of Internal Medicine, Erasmus MC, University Medical Centre Rotterdam, Rotterdam, The Netherlands; 36 Forth Valley Royal Hospital, Larbert, Scotland, UK; 37 Norfolk and Norwich University Hospitals NHS Foundation Trust, Norfolk and Norwich University Hospital, Norwich, UK; 38 Burnley General Hospital, Burnley, Lancashire, UK; 39 Head of Gastroenterology, University Hospital of Northern BC, Prince George, British Columbia, Canada; 40 Bradford Teaching Hospitals NHS Foundation Trust, Bradford Royal Infirmary, Bradford, UK; 41 Royal United Hospital Bath NHS Trust, Royal United Hospital, Avon, Bath, Somerset, UK; 42 Kettering General Hospital NHS Foundation Trust, Kettering General Hospital, Rothwell Road, Kettering, Northants, UK; 43 York Teaching Hospital NHS Foundation Trust, York, UK; 44 Luton and Dunstable University Hospital NHS Foundation Trust, Luton, Bedfordshire, UK; 45 Department of Thoracic and Upper Gastrointestinal Surgery, Royal Devon and Exeter NHS Foundation Trust, Exeter, UK; 46 County and Durham and Darlington NHS Foundation Trust, Bishop Auckland, County Durham, UK; 47 Guy’s and St Thomas’ NHS Foundation Trust, London, UK; 48 Barts Cancer Institute, Barts and The London School of Medicine and Dentistry, Queen Mary University of London, Charterhouse Square, London, UK; 49 Division of Public Health Sciences, Fred Hutchinson Cancer Research Centre, Seattle, Washington; 50 Department of Epidemiology, University of North Carolina School of Public Health, Chapel Hill, North Carolina; 51 Division of Research and San Francisco Medical Center, Kaiser Permanente Northern California, California; 52 Division of Gastroenterology and Hepatology, UNC School of Medicine, University of North Carolina, Chapel Hill, North Carolina; 53 Department of Oncology, The Medical School, University of Sheffield, UK; 54 Division of Epidemiology, University of Leeds, Leeds, UK; 55 Division of Human Biology, Fred Hutchinson Cancer Research Center, Seattle, Washington; 56 Department of Medical Epidemiology and Biostatistics, Karolinska Institutet, Stockholm, Sweden; 57 Princess Margaret Cancer Centre, Ontario Cancer Institute, Toronto, ON, Canada; 58 Division of Gastroenterology and Hepatology, Mayo Clinic, Rochester, Minnesota; 59 Registry, On behalf of the Romero; 60 Department of Population Sciences, Beckman Research Institute and City of Hope Comprehensive Cancer Center, Duarte, California; 61 Department of Preventive Medicine, University of Southern California/Norris Comprehensive Cancer Center, Los Angeles, California; 62 Department of Surgery, University of Saskatchewan, Saskatoon, Canada; 63 MRC Cancer Cell Unit, Hutchison-MRC Research Centre and University of Cambridge, Cambridge, UK; 64 Cancer Control, QIMR Berghofer Medical Research Institute, Queensland, Australia; 65 Department of Chronic Disease Epidemiology, Yale School of Public Health, New Haven, Connecticut; 66 Department of Biostatistics, University of Washington School of Public Health, Seattle, Washington; 67 Department of Gastroenterology, Tergooi Hospital, Hilversum, The Netherlands; 68 Department of Surgery, Erasmus MC, University Medical Centre Rotterdam, Rotterdam, The Netherlands; 69 Department of Gastroenterology and Hepatology, Academic Medical Centre, Amsterdam, The Netherlands; 70 Department of Genetics, University Medical Centre Groningen and University of Groningen, The Netherlands; 71 Department of Clinical Medicine & Institute of Molecular Medicine, Trinity Centre for Health Sciences, Trinity College Dublin, St James's Hospital, Dublin, Ireland; 72 Department of Surgery, Trinity Centre for Health Sciences, Trinity College Dublin, St. James’ Hospital, Dublin, Ireland; 73 Gastrointestinal Unit, Mater Misericordiae University Hospital, University College Dublin, Dublin, Ireland; 74 Faculty of Medicine, Imperial College London, South Kensington Campus, London, UK; 75 Faculty of Medicine, Imperial College, South Kensington Campus, London, UK; 76 University Hospitals Coventry & Warwickshire NHS Trust, Warwickshire, England; 77 Warwick Medical School, University of Warwick, Warwickshire, England

**Keywords:** EAC, Intestinal Metaplasia, Susceptibility, Cancer, ASE, allele-specific expression, BE, Barrett’s esophagus, BEACON, Barrett's and Esophageal Adenocarcinoma Consortium, CI, confidence interval, EAC, esophageal adenocarcinoma, eQTL, expression quantitative trait locus, GWAS, genome-wide association study, LD, linkage disequilibrium, OR, odds ratio, PC, principal component, SNP, single nucleotide polymorphism, TCGA, The Cancer Genome Atlas

## Abstract

**Background & Aims:**

Barrett's esophagus (BE) increases the risk of esophageal adenocarcinoma (EAC). We found the risk to be BE has been associated with single nucleotide polymorphisms (SNPs) on chromosome 6p21 (within the HLA region) and on 16q23, where the closest protein-coding gene is *FOXF1*. Subsequently, the Barrett's and Esophageal Adenocarcinoma Consortium (BEACON) identified risk loci for BE and esophageal adenocarcinoma near *CRTC1* and *BARX1*, and within 100 kb of *FOXP1*. We aimed to identify further SNPs that increased BE risk and to validate previously reported associations.

**Methods:**

We performed a genome-wide association study (GWAS) to identify variants associated with BE and further analyzed promising variants identified by BEACON by genotyping 10,158 patients with BE and 21,062 controls.

**Results:**

We identified 2 SNPs not previously associated with BE: rs3072 (2p24.1; odds ratio [OR] = 1.14; 95% CI: 1.09–1.18; *P* = 1.8 × 10^−11^) and rs2701108 (12q24.21; OR = 0.90; 95% CI: 0.86–0.93; *P* = 7.5 × 10^−9^). The closest protein-coding genes were respectively *GDF7* (rs3072), which encodes a ligand in the bone morphogenetic protein pathway, and *TBX5* (rs2701108), which encodes a transcription factor that regulates esophageal and cardiac development. Our data also supported in BE cases 3 risk SNPs identified by BEACON (rs2687201, rs11789015, and rs10423674). Meta-analysis of all data identified another SNP associated with BE and esophageal adenocarcinoma: rs3784262, within *ALDH1A2* (OR = 0.90; 95% CI: 0.87–0.93; *P* = 3.72 × 10^−9^).

**Conclusions:**

We identified 2 loci associated with risk of BE and provided data to support a further locus. The genes we found to be associated with risk for BE encode transcription factors involved in thoracic, diaphragmatic, and esophageal development or proteins involved in the inflammatory response.

Barrett’s esophagus (BE) is a common premalignant condition that affects up to 2% of the adult population in the Western world.^1^ BE comprises the second stage in the esophagitis–metaplasia–dysplasia–adenocarcinoma sequence. BE confers a 2%–4% lifetime risk of esophageal adenocarcinoma (EAC).^1^ Chronic gastric acid reflux is the predominant etiologic factor for BE. In addition, BE co-occurs with conditions such as intestinal metaplasia, hiatal hernia, obesity, and hypercholesterolemia.^2^, ^3^, ^4^, ^5^ Several factors, including the degree of acid reflux, hiatal hernia size, and the percentage of intestinal metaplasia–positive glands, can affect the progression to cancer. A role for genetics in the pathogenesis of gastroesophageal reflux disease, including BE and EAC, has been implicated on the basis of 3 observations: concordance in monozygous and dizygous twins^6^, ^7^, ^8^; the increased risk of disease in those with a positive family history^9^, ^10^; and, recently, the identification of single nucleotide polymorphisms (SNPs) associated with BE in Genome-Wide Association Studies (GWAS).^11^, ^12^ The proportion of variation in BE risk explained by common variants has been estimated to be 35%.^13^

Our GWAS previously identified 2 SNPs, on chromosomes 6p21 (rs9257809; *P* = 4.1 × 10^−9^) and 16q24 (rs9936833; *P* = 2.7 × 10^−10^), that are associated with BE.^11^ One of these loci lies within the HLA region and the other, close to *FOXF1*, which is involved in esophageal structure and development. Both SNPs have also been shown to be associated with risk of EAC.^14^ More recently, in a combined analysis of BE and EAC cases, the Barrett's and Esophageal Adenocarcinoma Consortium (BEACON) identified susceptibility SNPs within *CRTC1* and *BARX1*, and near *FOXP1*.^12^ The last 2 of these genes are known to be involved in esophageal development.^15^, ^16^

We aimed to identify further BE predisposition SNPs from our GWAS by performing wider and deeper independent replication of SNPs that already had promising disease associations.

## Methods

### Patient and Sample Collection Criteria

**Figure 1:**
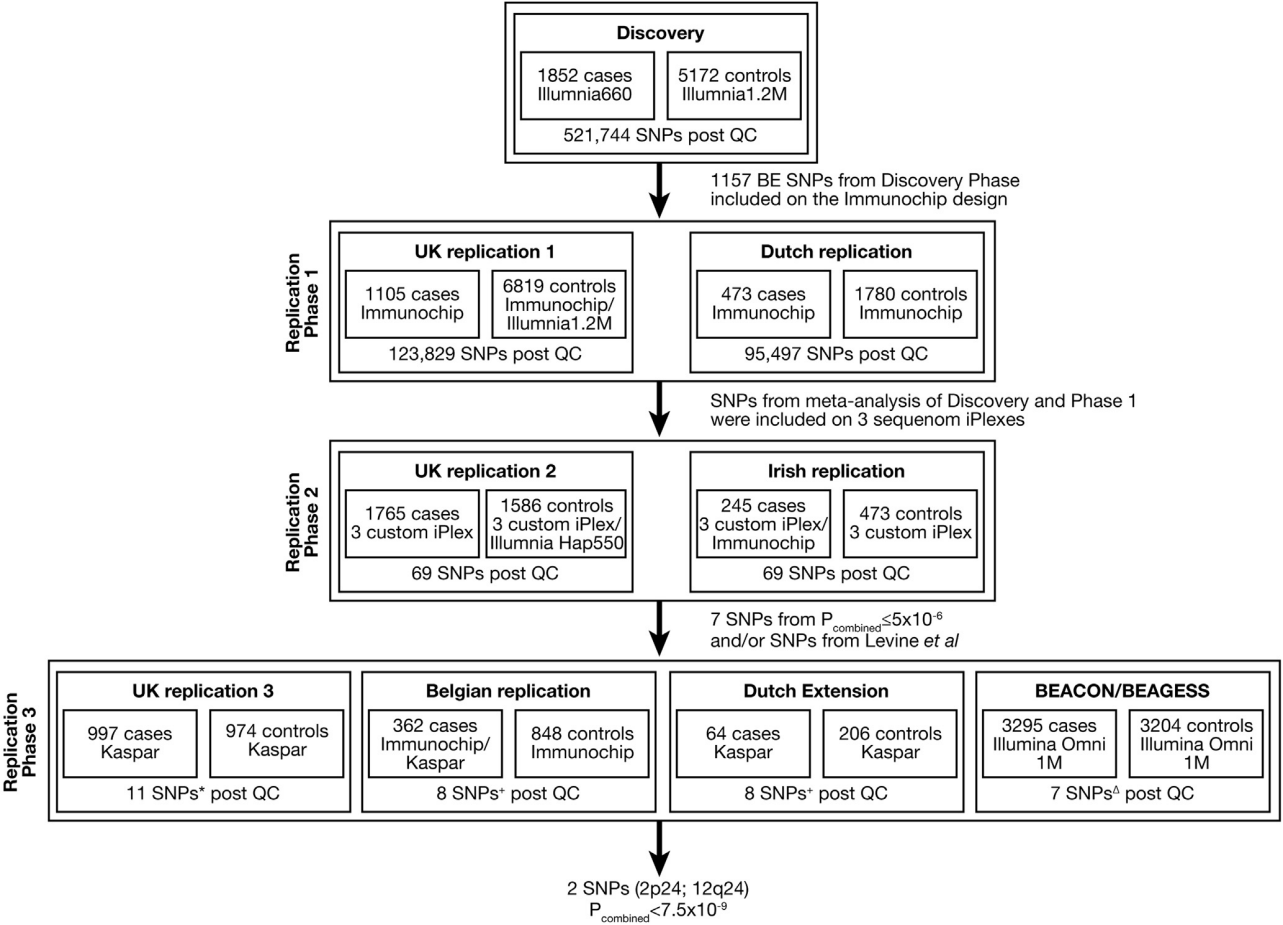
Outline of the phases of this study and the SNPs analyzed. Two SNPs described in Su et al^11^ had previously been genotyped in Replication Phase 2 and BEACON/BEAGESS samples. All other replication phase 3 samples are new to this study, as is the genotyping of additional SNPs in phases 2 and 3. Dutch Replication (Phase 1 Replication) and the Dutch extension (Phase 3 Replication) is one cohort in our analyses for the SNPs taken through to Replication Phase 3. ^∗^11 SNPs: Our SNPs: rs3072, rs6751791, rs2731672, rs2701108, rs189247, rs2043633, and rs12985909 and Levine et al^12^ SNPs: rs1497205, rs254348, rs3784262, and rs4523255. ^+^8 SNPs: Our SNPs: rs3072, rs6751791, rs2731672, rs2701108, rs189247, rs2043633, and Levine et al SNP: rs3784262. ^Δ^7 SNPs: Our SNPs: rs3072, rs6751791, rs2731672, rs2701108, rs189247, rs2043633.

Figure 1 outlines this study and the numbers of samples that contributed to each phase. As described previously,^11^ Discovery Phase cases were diagnosed with histologically confirmed BE and ascertained through the UK-based Aspirin Esomeprazole Chemoprevention Trial (AspECT) in Barrett's Metaplasia, a clinical trial of proton-pump inhibitor (esomeprazole) and aspirin as preventive agents for progression of BE to EAC.^17^ Replication Phase UK, Irish, Dutch, and Belgian patient samples were obtained from the Chemoprevention Of Premalignant Intestinal Neoplasia (ChOPIN) genetic study and the Esophageal Adenocarcinoma GenEtics (EAGLE) consortium.^1^ Replication Phase patients were diagnosed with BE with lengths of ≥1 cm (C1M1) circumferential disease or ≥2 cm tongue patterns (C0M2), according to the Prague criteria.^18^ Patient collection was in accordance with British Society of Gastroenterology criteria for BE^19^ and followed verification of endoscopic findings and proven BE on histopathologic records. Presence of EAC at presentation or subsequently was recorded, but was not an inclusion criterion.

### Sample Sets

#### Discovery Phase

BE patients (n = 1852) were UK participants in the AspECT study (Chief Investigator: Jankowski), HANDEL study (Chief Investigator: Jankowski), ChOPIN study (Chief Investigator: Jankowski), and population controls of white Caucasian origin (n = 5172) were from the common Wellcome Trust Case Control Consortium 2 (WTCCC2) set.^11^

#### Replication Phase 1

UK Replication 1 totaled 1105 BE patients from ChOPIN and 6819 controls. The controls comprised People of the British Isles (Chief Investigator: W. Bodmer) (n = 2578) and WTCCC2 (Chief Investigator: P. Donnelly) samples (n = 4241) that were not genotyped in the Discovery Phase. The Dutch replication samples consisted of 473 BE patients and 1780 controls from the University Medical Centre, Groningen. An additional 64 Dutch cases and 206 controls, provided since 2012 from Nijmegen and Rotterdam as part of EAGLE, were genotyped for the 7 SNPs taken into Replication Phase 3.

#### Replication Phase 2

UK Replication 2 comprised 1765 BE patients from the ChOPIN study. Controls (n = 1586) were from the Colorectal Tumour Gene Identification (CoRGI) Consortium^20^ (Chief Investigator: Tomlinson), comprising spouses or partners unaffected by cancer and without a family history (to 2^nd^-degree relative level) of colorectal neoplasia. All were of white UK ethnic origin. The Irish replication samples were 245 BE patients and 473 controls of white Caucasian origin from St James’s Hospital and Mater Misericordiae University Hospital, Dublin. Healthy donor controls were provided by Trinity Biobank.

#### Replication Phase 3

UK Replication 3 comprised 997 BE patients from the ChOPIN study and 974 female controls from the Genetics of Lobular Carcinoma In Situ in Europe (GLACIER) study (Chief Investigators: Sawyer, Roylance) with no personal or family history of breast cancer and of white Caucasian origin.^21^ The Belgian replication samples consisted of 362 cases and 848 controls from Leuven. Finally, 3295 BE patients and 3204 controls predominantly of northern European descent from the BEACON consortium GWAS (Chief Investigators: Vaughan, Whiteman, Levine) were included.^12^ All studies received ethical board approval (details in Supplementary Material). Two SNPs described in Su et al^11^ had been genotyped previously in Replication Phase 2 and BEACON/BEAGESS samples. All other Replication Phase 3 samples were new to this study.

### Genotyping

For all samples, genomic DNA was extracted from peripheral blood. Various genotyping methods were used, depending on the phase of the study and on pre-existing data from some sample sets. In brief, Discovery Phase genotyping was performed using the Illumina 660W-Quad array for cases and a custom Human 1.2M-Duo array for controls at the Wellcome Trust Sanger Institute.^11^ Replication Phase 1 genotyping was performed using the Illumina Immunochip at the Wellcome Trust Sanger Institute^11^ or as described in Trynka et al.^22^

In Replication Phase 2, samples underwent custom genotyping for SNPs that met one of the following criteria: *P*_*association*_ < 10^−4^ in combined Discovery and Replication Phase 1 analysis (n = 63); *P*_*association*_ < 10^−4^ in Discovery Phase, but not included in Replication Phase 1 (n = 12); and *P*_*association*_ < 10^−4^ in a sex-stratified analysis of the Discovery phase (n = 5); and candidate polymorphisms previously reported as associated with BE and not well tagged by the Discovery Phase or Immunochip arrays, specifically, *MSR1* p.Arg293Gly,^23^ and variants in *IGF1R* and *GHR*^24^ (Supplementary Table 1). Sequenom iPLEX assays were successfully designed for 65 of these SNPs and genotyping was performed at the Wellcome Trust Sanger Institute. Genotypes were assigned using MassArray TyperAnalyzer 4.0 (Sequenom). Samples with sex discrepancies between manifests or with overall call rates <95% were excluded, as were SNPs with call rates of <95%. Where a SNP was in the top 40 of the prioritized SNPs (by *P* value) and had failed at the design stage of the iPLEX (n = 3), genotyping was performed by KASPar in the full Replication Phase 2 sample set. Seventy-seven of two hundred and forty-five Irish cases passing quality control were genotyped using iPLEX assays, and the other cases were genotyped using the Immunochip. Eighteen iPLEX SNPs were not analyzed in the Irish cohort, as the SNPs were not present on the Immunochip. The samples were also genotyped for 4 SNPs after publication of Levine et al.^12^

Replication Phase 3 samples (Supplementary Table 2) were genotyped using KASPar for 7 SNPs prioritized after analysis of the previous phases. The samples were also genotyped for 4 SNPs after publication of Levine et al.^12^ All SNPs had call rates >95%. Sample exclusions were as for Replication Phase 2.

We had previously demonstrated >99% concordance between genome-wide array, Immunochip, and KASPar assays for other SNPs.^11^ Sequenom call rate was >96%. For samples analyzed only by KASPar, genotyping QC was tested using duplicate DNA samples within studies and SNP assays, together with direct sequencing of subsets of samples to confirm genotyping accuracy. For all SNPs, >98% concordant results were obtained.

### Association Analysis

Case-control analysis was performed using frequentist tests under a missing data logistic regression model, as implemented in SNPTEST (version 2.4.1). Principal component analysis was performed for all samples typed on GWAS arrays (Discovery Phase) and has been described in Su et al.^11^ As described previously, principal component 1 (PC1) was included as a covariate in all analyses of the Discovery Phase. Each SNP was tested as a quantitative explanatory variable, coded as 0, 1, 2. We used GWAMA (version 2.1) to implement fixed inverse variance-based methods for meta-analysis.^25^ The software tests for heterogeneity of effects between studies^26^ and enables sex-specific meta-analysis.

### Replication and Validation of Single Nucleotide Polymorphisms From BEACON/BEAGESS Meta-analysis

In order to examine the 4 genome-wide significant BE + EAC SNPs and 83 other SNPs with *P*_*assoc*_ < 10^−4^ in the BEACON/BEAGESS data, we performed association testing using AspECT Discovery Phase cases. Because our Discovery Phase controls overlapped with those used by Levine et al,^12^ we used 1898 white European controls from colorectal cancer GWAS studies CoRGI and Colon Cancer Family Registry.^27^ Genotypes were imputed where necessary, with strict cut-offs for imputation quality.^28^, ^29^

### Other Analyses

Details of imputation, fine mapping, pathway analyses, estimation of heritability, and URLs are provided in the Supplementary Material.

## Results

### Identification of Two New Barrett’s Esophagus Predisposition Single Nucleotide Polymorphisms

In order to identify further loci associated with BE, we prioritized 65 SNPs (Supplementary Table 1) with the best evidence of association with BE from our previous GWAS Discovery Phase and Replication Phase 1 (details in Methods).^11^ These SNPs were genotyped in an additional 1765 cases and 1586 controls from the UK and in an Irish cohort of 245 cases and 473 controls (Replication Phase 2, previously used to genotype rs9257809 and rs9936833 described in Su et al^11^). After meta-analysis of these new data together with Discovery Phase and Replication Phase 1, seven SNPs showing evidence of associations with BE risk at *P*_meta_ <5 × 10^−6^ were identified and genotyped in Replication Phase 3 samples (Table 1). After Replication Phase 3, two SNPs—rs3072 and rs2701108 on chromosome 2p24 and 12q24, respectively—reached the level of significance conventionally used for GWAS (*P* = 5 × 10^−8^) (Table 1). Combined *P*_meta_ values were 1.8 × 10^−11^ for rs3072 (OR = 1.14; 95% CI: 1.09–1.18) and 7.5 × 10^−9^ for rs2701108 (OR = 0.90; 95% CI: 0.86–0.93), derived from a total sample of 10,158 BE cases and 21,062 controls (Supplementary Table 3). The associations remained at or near genome-wide significance upon restricting the analysis to the 8521 cases with histologically proven intestinal metaplasia (rs3072: *P* = 1.3 × 10^−9^; OR = 1.13; 95% CI: 1.09–1.17; rs2701108: *P* = 6.2 × 10^−8^; OR = 0.90; 95% CI: 0.86–0.94). There was no evidence of sex heterogeneity for either SNP (Supplementary Table 4).

**Table 1:** Meta-analysis of Discovery and Replication Phase Sample Sets for SNPs Taken Into Replication Phase 3

SNP	Chr	Position (build 37)	Minor/ major	Discovery MAF (cases /controls)	Discovery	Replication phase 1 (rep 1)	Meta 1 (discovery + rep 1)	Replication phase 2 (rep 2)	Meta 2 (meta 1+ rep 2)	Replication phase 3 (rep 3)	Final meta (meta 2 + rep 3)	Final meta I^2^	N
**rs3072**	**2**	**20878406**	**G/A**	**0.41/0.36**	**1.23** **(1.14–1.33)** **2.64 × 10^−7^**	**1.10** **(1.02–1.19)** **1.33 × 10^−2^**	**1.16** **(1.10–1.23)** **8.13 × 10^−8^**	**1.13** **(1.03–1.24)** **7.00 × 10^−3^**	**1.16** **(1.10–1.21)** **2.27 × 10^−9^**	**1.11** **(1.04–1.17)** **1.02 × 10^−3^**	**1.14** **(1.09–1.18)** **1.75 × 10^−11^**	**0.42**	**8**
rs6751791	2	35581997	A/G	0.51/0.48	1.15 (1.06**–**1.23) 5.03 **×** 10^**−**4^	1.16 (1.07**−**1.25) 1.45 **×** 10^**−**4^	1.15 (1.09**−**1.21) 2.68 **×** 10^**−**7^	1.07 (0.97**−**1.16) 1.64 **×** 10^−1^	1.13 (1.08**−**1.18) 2.91 **×** 10^**−**7^	0.99 (0.93**−**1.05) 7.99 **×** 10^**−**1^	1.08 (1.04**−**1.12) 7.65 **×** 10^**−**5^	0.60	8
rs2731672	5	176842474	A/G	0.27/0.24	1.18 (1.09**−**1.28) 1.64 **×** 10^**−**4^	1.14 (1.04**−**1.24) 3.87 **×** 10^**−**3^	1.16 (1.09**−**1.23) 2.54 **×** 10^**−**6^	1.09 (0.99**−**1.21) 8.20 **×** 10^−2^	1.14 (1.08**−**1.20) 8.33 **×** 10^**−**7^	0.95 (0.89**−**1.02) 1.81 **×** 10^**−**1^	1.07 (1.03**−**1.12) 1.66 **×** 10^**−**3^	0.63	8
**rs2701108**	**12**	**114674261**	**G/A**	**0.38/0.41**	**0.88** **(0.81–0.95)** **1.00 × 10^−3^**	**0.87** **(0.81–0.94)** **4.40 × 10**^**−4**^	**0.87** **(0.83–0.92)** **1.51 × 10**^**−6**^	**0.89** **(0.81–0.97)** **1.10 × 10^−2^**	**0.88** **(0.84–0.92)** **5.68 × 10^−8^**	**0.93** **(0.87–0.99)** **1.42 × 10^−2^**	**0.90** **(0.86–0.93)** **7.48 × 10^−9^**	**0.14**	**8**
rs189247	15	97586630	A/G	0.41/0.37	1.18 (1.09**−**1.27) 5.67 **×** 10^**−**5^	1.14 (1.05**−**1.23) 1.25 **×** 10^**−**3^	1.15 (1.09**−**1.22) 2.91 **×** 10^**−**7^	1.10 (1.00**−**1.20) 4.90 **×** 10^−2^	1.14 (1.09**−**1.19) 6.12 **×** 10^**−**8^	0.96 (0.90**−**1.02) 1.73 **×** 10^**−**1^	1.10 (1.06**−**1.14) 3.55 **×** 10^**−**7^	0.20	8
rs2043633	16	5819274	C/A	0.37/0.41	0.85 (0.79**−**0.92) 6.04 **×** 10^**−**5^	0.88 (0.82**−**0.95) 9.80 **×** 10^**−**4^	0.87 (0.82**−**0.92) 2.49 **×** 10^**−**7^	0.88 (0.80**−**0.96) 5.00 **×** 10^**−**3^	0.87 (0.83**−**0.91) 4.74 **×** 10^**−**9^	0.99 (0.94**−**1.05) 8.39 **×** 10^**−**1^	0.92 (0.88**−**0.95) 2.25 **×** 10^**−**6^	0.58	8
rs12985909	19	18439383	G/A	0.48/0.45	1.12 (1.04**−**1.21) 2.94 **×** 10^**−**3^	1.12 (1.04**−**1.21) 2.73 **×** 10^**−**3^	1.12 (1.06**−**1.18) 2.45 **×** 10^**−**5^	1.11 (1.01**−**1.21) 2.70 **×** 10^**−**2^	1.12 (1.07**−**1.17) 1.99 **×** 10^**−**6^	1.07 (1.01**−**1.13) 2.63 **×** 10^**−**2^	1.10 (1.06**−**1.14) 3.28 **×** 10^**−**7^	0.00	8

NOTE. For each phase, association data show (top to bottom) OR, (95% CI), and *P*_assoc_. Results are presented with respect to the minor allele. rs3072 and rs2701108 reached genome-wide significance and thus are shown in bold. In BEACON, rs7598399 was used as a proxy for rs6751791 (*r*^2^ = 1) and rs189247 was imputed from 4 genotyped SNPs (rs991757, rs2670927, rs2670930, and rs234540) with accuracy approximately 98%. The Dutch extension samples were analyzed with the previously described Dutch replication samples as part of Rep 1. The *P* value threshold for including a SNP in Phase 2 was 1 × 10^−4^ and that for inclusion in Phase 3 was 5 × 10^−6^.

Chr, chromosome; I^2^, I^2^ heterogeneity index; MAF, minor allele frequency; N, number of studies.

#### In Silico Fine Mapping and Annotation of the Chromosome 2p24 and 12q24 Loci

**Figure 2:**
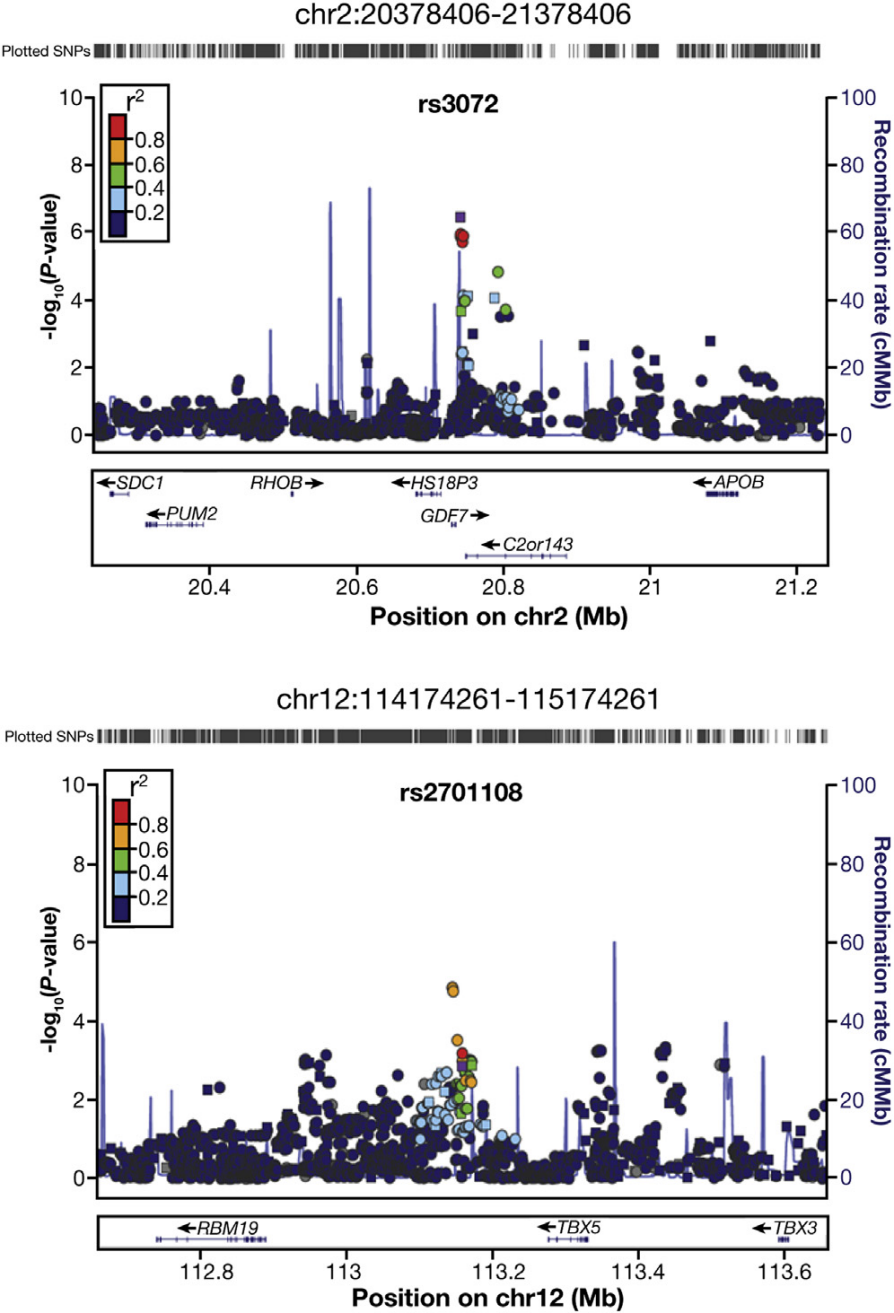
Regional plots of association (*left y-axis*) and recombination rates (*right y-axis*) for the chromosomes 2p24 and 12q24 loci after imputation. The lead genotyped SNP is marked with a *purple square*. Imputed SNPs are plotted as *circles* and genotyped SNPs as *squares*.

rs3072 lies between 2 genes, mapping 7.5 kb downstream of *GDF7* (also known as *BMP12*) and 6.5 kb downstream of *C2orf43* (Figure 2). rs2701108 is 117 kb downstream of *TBX5* and 270 kb upstream of *RBM19*. We imputed in our Discovery Phase all SNPs in 1-Mb regions flanking each of the lead SNPs. At chromosome 2p24, rs3072 remained the most strongly associated SNP, but at chromosome 12q24, rs1920562 was more strongly associated with disease risk (*P*_*Discovery*_ = 1.4 × 10^−5^; OR = 0.84) than the lead genotyped SNP (*P*_*Discovery*_ = 1.4 × 10^−3^; OR = 0.88). rs1920562 (linkage disequilibrium [LD] with rs2701108; *r*^2^ = 0.6) lies 131 kb downstream of *TBX5* and 256 kb upstream of *RBM19*. Nonsynonymous SNPs in the genes flanking the signals on chromosomes 2 and 12 were not in strong LD (*r*^2^ < 0.4; D’ < 0.8) with the lead genotyped or imputed SNPs, suggesting that the functional variants may have effects on gene expression and regulation rather than protein sequence. Haploregv2^30^ and Annovar^31^ were used to annotate SNPs in strong LD (*r*^2^ > 0.4) with the 2 lead tagging SNPs.

rs3072, which may alter a GATA binding motif, lies within a region of histone modifications, such as H3K4Me1, which mark enhancers (data from lymphoblastoid cell line (LCL) GM12878). Three other SNPs in LD with rs3072 map to the enhancer region detected in GM12878. One of these, rs7255, maps to a site of high evolutionary conservation/constraint; another SNP, rs9306894, whilst not at a conserved site (Supplementary Table 5), is predicted as “likely to affect protein binding and linked to expression of a gene target” according to RegulomeDB.^32^ We examined associations between SNPs in this region and gene expression in The Cancer Genome Atlas (TCGA) EAC data.^33^ Genotypes were only available for rs9306894 in the chromosome 2 locus and gene expression data had been obtained using RNASeq. After correcting for copy number, we determined associations between rs9306894 genotype and total RNA levels for expression quantitative trait locus (eQTL) analysis and bias in allelic expression of coding SNPs (allele-specific expression [ASE] analysis). There was no significant association with expression of the closest genes *GDF7, HS1BP3* and *C2orf43* (*P*_eQTL_ > .20; *P*_ASE_ > .38; n = 62) and no genome-wide association with expression of any other gene was present (*q* > .05, details not shown). In public data sets based on monocytes^34^ and on lymphoblastoid cell lines and adipose tissue,^35^ *C2orf43* is the suggested target of rs9306894 following eQTL studies (GENevar; *P* = 7 × 10^−4^). rs9306894 genotype was not associated with *GDF7* expression in these cell types.

rs2701108 itself is not likely to be a functionally regulatory SNP, but rs1920562, which showed the strongest regional association after imputation, is a more promising candidate (Supplementary Table 6). This SNP maps to a highly conserved base and a region containing enhancer marks in human embryonic stem cells (h1-ESC) and lung fibroblasts (NHLF). rs1920562 and an additional SNP (rs1247938) in moderate LD (*r*^2^ = 0.52) with rs2701108, are highlighted by Regulome DB as being the most likely SNPs in this region to affect protein binding. CTCF and RAD21 binding are predicted to be affected by rs1247938 and the ability of IKZF1 to bind is predicted to be altered by rs1920562. Expression analyses were performed for the rs2701108 region, in the same way as for rs9306894. However, none of the three rs2701108 region SNPs was associated with *TBX5, TBX3* or *RBM19* expression in the TCGA data (*P*_eQTL_ > .39; *P*_ASE_ > .43; n = 62), was an eQTL in whole-transcriptome analysis, or was an eQTL in the public databases (details not shown).

### Pathway/Geneset Enrichment Analysis

Improved Gene Set Enrichment Analysis for Genome Wide Association Study (iGSEA4GWAS) and SNP ratio test respectively found 26 and 34 pathways significantly enriched in cases at False Discovery Rate–corrected *P* < .05. Genetic Genomics Analysis of Complex Data (Gengen) did not identify any pathways with corrected *P* < .05, but 10 pathways had *P* < .25 (Supplementary Table 7). Three pathways (type 1 diabetes mellitus, KEGG antigen processing and presentation, and KEGG autoimmune thyroid disease) were identified by all methods and the SNPs mapping to each pathway were subjected to set-based association tests using PLINK, producing empirical *P* values of .0021, .025, and .0317, respectively. The SNPs within these pathways that showed replication at *P* < .05 in Replication Phase 1 all mapped to chromosome 6p21, either close to or within HLA genes. Upon removal of the HLA genes from all the pathways, 20/26 pathways originally with False Discovery Rate *P* < .05 according to the iGSEA4GWAS approach remained significant, but only 1/34 pathways identified by SNP ratio test remained significant. No HLA-depleted pathways were even suggestive of enrichment (all *P* > .25) by Gengen. The top networks identified by Ingenuity Pathway Analysis were Cardiovascular System Development and Function, Embryonic Development and Organ Development (Supplementary Figure 1). The 5 genes implicated by the BE susceptibility SNPs here are all involved in development at a cellular, embryonic, organ, and organism level. Bone morphogenetic protein 4 was the most significant upstream regulator (*P*_*overlap*_ = 1.99 × 10^−6^).

### Barrett’s Esophagus Heritability

Genome-wide haplotype-tagging SNP data on the 1852 cases and 5172 controls in the Discovery Phase of this study were used in Genome-wide Complex Trait Analysis to estimate the proportion of variation in risk of BE that can be explained by common genetic variants. In line with our previous, disease score test analysis,^11^ we found a statistically significant component of BE risk to be polygenic (9.99% [SE 1.2%]). This was a lower estimate than that recently derived by Ek et al.^13^

### Replication Testing of Previously Reported Barrett’s Esophagus Susceptibility Single Nucleotide Polymorphisms at Candidate Loci

Using a systematic review, we identified 26 polymorphisms reported in the literature to be associated with BE (Supplementary Table 8). In our Discovery Phase samples, 20 of 26 SNPs were directly genotyped or were in strong LD (*r*^2^ > 0.7) with a directly genotyped SNP. Only one of these SNPs showed a nominally significant association (*P* < .05) in our data (rs909253, proxy for rs1041981, *r*^2^ = 0.93; OR = 1.12; *P* = .005). This SNP was also present on the Immunochip and showed additional evidence of replication (*P*_meta_ = 3.1 × 10^−4^, OR = 1.07). rs909253 maps to a highly conserved base (based on SiPhy score) in an intron of *LTA* (tumor necrosis factor–β) where histone marks associated with both promoters and enhancers are present in lymphoblastoid cell lines (LCLs).^30^ PBX3, PU1, POL2, YY1, and nuclear factor–κB have all been found to bind here in ENCODE ChIP-seq experiments. No data were available for this SNP in RegulomeDB. We were able to genotype 3 other candidate SNPs previously reported for BE susceptibility (rs41341748, rs2715425, rs6898743) on the Sequenom iPLEX panel used to genotype UK Replication 2. None of these SNPs showed associations with BE risk (Supplementary Table 1). Because rs41341748 (*MSR1* p.Arg293Gly) has a low minor allele frequency (<5%) and consequently our power to detect an association was relatively low, we additionally genotyped it in UK Replication 3. After meta-analysis of Replication 2 and 3, we remained unable to replicate the previously reported association^23^ between this SNP and BE risk (OR = 1.07; 95% CI: 0.70–1.43; *P* = .79).

### Assessment of Previously Reported Barrett’s Esophagus + Esophageal Adenocarcinoma Single Nucleotide Polymorphisms and Meta-analysis With BEAGESS Data

Three new genome-wide significant BE + EAC loci (4 SNPs) were recently identified by Levine et al^12^ in a combined analysis of EAC and BE: rs10419226 and rs10423674 in *CRTC1*, rs11789015 in *BARX1* and rs2687201 within 100 kb of *FOXP1.* None of these associations was genome-wide significant at *P* < 5 × 10^−8^ when Levine et al restricted their analysis to BE cases alone, although one SNP, rs10419226, within *CRTC1*, reached *P* = 5.5 × 10^−8^. In our datasets, only rs10423674 had been directly genotyped, but the remaining SNPs were all reliably imputed in our Discovery Phase samples (Info scores >0.95). However, the controls used in the replication phase of the Levine et al^12^ study overlapped entirely with the controls used in our Discovery phase (WTCCC2 controls) and we therefore used alternative UK controls from the CoRGI study (see Methods).

Of the 4 Levine SNPs, rs10423674, 1 of 2 SNPs in *CRTC1*, and rs2687201, near *FOXP1*, were supported in this study (*P* = 0.02; OR = 1.14; 95% CI: 1.03–1.27 and *P* = 0.05; OR = 0.94; 95% CI: 0.88–1.00, respectively). There was also some support for rs11789015, near *BARX1* (*P* = 0.07; OR = 0.90; 95% CI: 0.81–1.01). However, the association at rs10419226, within *CRTC1*, was not replicated in our data (*P* = 0.87; OR = 1.01; 95% CI: 0.91–1.11). All 4 SNPs still reached genome-wide significance (*P* < 5 × 10^−8^) upon meta-analysis of our BE and Levine’s BE + EAC datasets. In a BE-only meta-analysis, the associations improved with the inclusion of our data for 3 out of the 4 SNPs, with 1 (rs2687201, *FOXP1*) reaching genome-wide significance for BE (Table 2).

**Table 2:** Meta-analysis With Our Data for 4 BE/EAC SNPs and 4 Other Selected SNPs with *P* < 1 × 10^−4^ from Levine et al^12^

SNP	Chr	Position	Nearby genes	Minor/ major allele	BE+/−EAC	Levine et al meta OR (95% CI)	*P*_*Levine*__*et al*_	This study meta OR (95% CI)	*P*_*This Study*_	This study + Levine et al^12^ meta OR (95% CI)	*P*_meta_	Meta I^2^	No. of studies
rs2687201	3	70928930	FOXP1	T/G	BE	1.18 (1.10–1.26)	2.00 × 10^−6^	1.14 (1.03–1.27)	1.18 × 10^−2^	1.16 (1.10−1.23)	4.61 × 10^−8^	0.00	3
					BE/EAC	1.18 (1.12−1.25)	5.47 × 10^−9^			1.17 (1.11−1.23)	6.70 × 10^−10^	0.00	3
rs11789015	9	96716028	BARX1	G/A	BE	0.85 (0.79−0.91)	5.08 × 10^−6^	0.90 (0.81−1.01)	6.63 × 10^−2^	0.86 (0.81−0.92)	1.38 × 10^−6^	0.00	3
					BE/EAC	0.83 (0.79−0.88)	1.02 × 10^−9^			0.85 (0.81−0.89)	1.14 × 10^−10^	0.00	3
rs10419226	19	18803172	CRTC1	A/C	BE	1.19 (1.12−1.26)	5.54 × 10^−8^	1.01 (0.91−1.11)	8.65 × 10^−1^	1.13 (1.08−1.20)	2.14 × 10^−6^	0.82	3
					BE/EAC	1.18 (1.12−1.24)	3.55 × 10^−10^			1.14 (1.09−1.19)	1.17 × 10^−8^	0.82	3
rs10423674	19	18817903	CRTC1	T/G	BE	0.85 (0.80−0.91)	1.92 × 10^−6^	0.94 (0.88−1.00)	4.88 × 10^−2^	0.89 (0.85−0.93)	2.99 × 10^−7^	0.40	5
					BE/EAC	0.84 (0.80−0.89)	1.75 × 10^−9^			0.88 (0.84−0.91)	4.87 × 10^−11^	0.49	5
rs1497205	4	76169067	PARM1, RCHY1	C/T	BE	0.86 (0.80−0.92)	2.86 × 10^−5^	0.92 (0.87−0.98)	7.59 × 10^−1^	0.90 (0.86−0.94)	2.57 × 10^−6^	0.00	6
					BE/EAC	0.87 (0.82−0.93)	1.28 × 10^−5^			0.90 (0.86−0.94)	3.68 × 10^−7^	0.00	6
rs254348	16	65980789		T/C	BE	0.88 (0.83−0.94)	1.15 × 10^−4^	0.95 (0.91−1.01)	8.88 × 10^−2^	0.93 (0.89−0.97)	5.49 × 10^−4^	0.53	6
					BE/EAC	0.89 (0.84−0.94)	1.40 × 10^−5^			0.92 (0.89−0.96)	2.81 × 10^−5^	0.53	6
rs3784262	15	58253106	ALDH1A2	G/A	BE	0.85 (0.80−0.90)	3.62 × 10^−7^	0.93 (0.89−0.98)	5.13 × 10^−3^	0.91 (0.87−0.94)	1.37 × 10^−6^	0.12	9
					BE/EAC	0.88 (0.83−0.92)	6.72 × 10^−7^			0.90 (0.87−0.93)	3.72 × 10^−9^	0.16	9
rs4523255	8	8713038	MFHAS1	A/G	BE	1.13 (1.06−1.21)	2.46 × 10^−4^	1.07 (1.01−1.12)	2.11 × 10^−2^	1.09 (1.05−1.14)	2.48 × 10^−5^	0.36	6
					BE/EAC	1.13 (1.07−1.20)	4.15 × 10^−5^			1.09 (1.05−1.14)	9.24 × 10^−6^	0.46	6

NOTE. The minimum meta-analysis comprised the Levine et al^12^ Discovery and Replication Phases and our Discovery Phase (with amended controls, as described in Methods). rs10423674 was additionally genotyped in our UK and Dutch Replication Phase 1. rs1497205, rs254348, rs3784262, and rs4523255 were genotyped in our UK and Dutch Replication Phase 1 and UK Replication Phase 2. rs3784262 was also genotyped in Irish Replication Phase 2 samples and UK and Belgium Replication Phase 3 samples. For rs10419226, which shows evidence of inter-study heterogeneity, random effects model *P* values for BE and BE/EAC are .10 and .04, respectively.

Chr, chromosome.

We then addressed the other 87 other SNPs with *P*_*assoc*_ < 10^−4^ in the Levine data (Supplementary Table 3 of Levine et al^12^). Of these, 73 were directly genotyped in our samples or could be imputed with an IMPUTE2 info score of >0.95. Of the 10 SNPs that could not be imputed with high quality, only one had *P*_*assoc*_ < 10^−5^ in the original Levine data; we therefore genotyped this SNP (rs11771429) using KASPar in our cases and controls. We did not obtain genotypes for the remaining 9 SNPs. On performing a meta-analysis of the Levine BE + EAC cases with our UK Discovery Phase, 4 SNPs (rs1497205, rs254348, rs3784262, and rs4523255) showed *P*_*meta*_ < 10^−5^ and were not strongly correlated with 1 of the 4 BE + EAC SNPs reported previously. We therefore genotyped these 4 SNPs in our Replication Phase samples; rs3784262 (within *ALDH1A2*) was associated with BE + EAC (OR = 0.90; 95% CI: 0.87–0.93; *P* = 3.72 × 10^−9^). No SNP was formally associated with BE alone (Table 2). eQTL and ASE analysis (see Results - In Silico Fine Mapping and Annotation of the Chromosome 2p24 and 12q24 Loci) did not show associations for the rs3784262 proxy rs7165247 in TCGA data or other public data sets (details not shown).

## Discussion

We have added 2 new BE predisposition SNPs, rs3072 on chromosome 2p24 and rs2701108 on chromosome 12q24, to the 2 BE SNPs on chromosome 6p21 (HLA region) and chromosome 16q23 (near *FOXF1*) that we reported previously.^11^ Both of the new SNPs remained at or very near genome-wide significance when analysis was restricted to cases with intestinal metaplasia. In silico fine mapping provided evidence that rs3072 and/or 1 of 3 nearby SNPs might be functional because they map to putative enhancer regions. The nearby gene, *GDF7*, is the best functional candidate, because this encodes the BMP12 protein and the BMP pathway has previously been implicated in the development of BE.^36^ The importance of this pathway in BE is also suggested in Ingenuity Pathway Analysis, where bone morphogenetic protein 4 acts upstream of proteins encoded by genes close to the BE predisposition SNPs. *GDF7* plays a role in the neural system and tendon/ligament development and repair,^37^, ^38^ and also regulates Hedgehog and Wnt signaling pathways that impact on esophageal development through *FOXF1* and *TBX5*. In the chromosome 12q24 region, rs1920562 (the top imputed SNP) provided the strongest association signal and maps to a possible enhancer. Gene expression analysis did not suggest the target of the chromosome 12q24 variation, although *TBX5* is a very strong functional candidate. It is involved in cardiac development and its deficiency causes thoracic malformations and abnormalities of the diaphragmatic musculature,^39^, ^40^ which could predispose patients to hiatus hernia and acid reflux, 2 subphenotypes of BE.

Messenger RNA expression analysis using TCGA EAC data and public data from leukocytes and adipocytes provided little evidence that rs3072 or rs2701108 (or other SNPs in strong LD) were eQTLs or influenced ASE. The absence of these associations is typical for GWAS or cancer or precancerous traits. Even for the “prototypic” multicancer SNP rs6983267, convincingly demonstrating the effects of SNP alleles on gene expression has required considerable additional work in a variety of systems, and even now, consistent eQTL and ASE associations have not been shown.^41^, ^42^, ^43^, ^44^ The likely major reason for the lack of eQTLs at GWAS SNPs is that the SNPs have their effects in a restricted set of cells or at a particular time. There is, for example, evidence that the forkhead box (FOX) proteins are most strongly expressed during embryogenesis, and that the levels of these transcription factors are critical for proper development.^45^, ^46^, ^47^ Given this, our first choice in searching for eQTLs would be cells in the developing human thorax. Unfortunately, such sample collections do not currently exist.

We showed rs2687201 (*FOXP1*) to be associated with disease in a BE-only analysis. Our data generally support the report by Levine et al^12^ of associations between BE + EAC and SNPs on chromosome 3 (*FOXP1*), chromosome 9 (*BARX1*), and one of the SNPs on chromosome 19 (*CRTC1*). However, we were not able to replicate the association observed for another SNP (rs10419226) in *CRTC1*. For this last SNP, the meta-analysis showed evidence of significant heterogeneity between the BEACON/BEAGESS data and our data (Table 2), and in the absence of clear reasons for this difference, we caution against drawing firm conclusions here.

We found another SNP, rs3784262 (*ALDH1A2*), to be formally associated with BE + EAC upon meta-analysis of our data with the Levine BE + EAC dataset. *ALDH1A2* encodes retinaldehyde dehydrogenase 2, which catalyzes the synthesis of retinoic acid and may also be involved in alcohol metabolism.^48^ Of the candidate SNPs we assessed (Supplementary Table 8), we found supporting evidence, albeit short of genome-wide significance, for rs909253 (*P* = 3.1 × 10^−4^), mapping to an intronic region of *LTA* within the HLA region, but not in LD with rs9257809, the other HLA BE SNP.

We previously reported that our original GWAS provided evidence that multiple common variants, each with small effects contribute to BE susceptibility.^11^ Ek et al^13^ recently estimated that the heritability of BE is 35% (SE 6%). We also found that the heritability of BE is highly significant, but explains only 9.99% of BE risk (SE 1.2%). Our GWAS consisted of UK cases and controls, and the BEACON/BEAGESS samples used by Ek et al originated from 3 continents (Europe, North America, and Australia). Cryptic population stratification could perhaps explain the larger estimate of heritability obtained using the BEACON/BEAGESS GWAS. In addition, we used software to calculate LD-adjusted kinships, such that the SNPs used in the heritability analysis were weighted according to local LD structure. It has been found that heritability estimation from genome-wide SNPs is highly sensitive to uneven LD; causal SNPs in regions of high LD can lead to overestimation of heritability and conversely causal SNPs in regions of low LD can result in an underestimation of heritability.^49^

Although the BE GWAS have not yet identified the functional SNPs in each region or their gene targets, the information generated already permits the generation of hypotheses regarding processes that may be involved in BE. First, transcription factors involved in development and structure of the thorax, diaphragm, and esophagus may be important: the SNPs near *FOXF1*, *FOXP1*, *BARX1*, and *TBX5* might act in this way and the genes appear to be functionally related (Supplementary Figure 1). Second, the inflammatory response may be important: the SNPs within the HLA region (rs9257809 and, perhaps, rs909253) might act in this way and pathway analysis provided suggestive evidence of a role for type 1 diabetes genes in BE etiology. A plausible, testable hypothesis is that these 2 groups of SNPs respectively influence the tendency to gastroesophageal reflux disease, perhaps through thoracic and diaphragmatic structure (hiatal hernia defect), and the inflammatory response to the refluxed gastric acid. Given the limited scope for clinical intervention in the former processes, we await with interest the outcome of trials such as AspECT that target the inflammatory response to gastric reflux.^1^, ^17^
