# Supplementary material for: Polymorphisms Near TBX5 and GDF7 Are Associated With Increased Risk for Barrett’s Esophagus
Source: Gastroenterology. 2015 Feb;148(2):367–78. doi: 10.1053/j.gastro.2014.10.041 (PMC4315134; doi:10.1053/j.gastro.2014.10.041)
Supplement: Supplementary Data [file mmc1.pdf]

Supplementary Table 1. SNPs Prioritized for Additional Genotyping in Stage 2 Samples (Irish Cohort of 245 Cases and 473 Controls and a UK Cohort of 1765 Cases and 1586 Controls)

SNP	Chr	Position (build 36)	Priority	Sequenom design successful	Sequenom call rate >95%	Reason for inclusion	Meta of UKREP2 and Irish cohorts OR (95% CI)	Meta of UKREP2 and Irish cohorts *P* value	No. of cohorts genotyped	Meta of discovery and replication phases 1 and 2 OR (95% CI)	Meta of discovery and replication phases 1 and 2 *P* value
rs41341748	8	16056965	1	Yes	1	*MSR1* candidate variant	1.09 (0.64–1.54)	.701	1	1.09 (0.64–1.54)	.701
rs3072	2	20741887	2	Yes	1	meta $P < 1 \times 10^{-4}$	1.13 (1.04–1.23)	.007	5	1.15 (1.11–1.2)	4.04×10^{-9}
rs6751791	2	35435501	3	Yes	0.998	meta $P < 1 \times 10^{-4}$	0.94 (0.85–1.03)	.164	5	0.88 (0.84–0.93)	1.82×10^{-7}
rs10083033	12	1.04E+08	4	Yes	1	meta $P < 1 \times 10^{-4}$	1.00 (0.91–1.09)	.943	5	0.90 (0.85–0.94)	5.82×10^{-6}
rs189247	15	95387634	5	Yes	0.999	meta $P < 1 \times 10^{-4}$	0.91 (0.82–1.00)	.049	5	0.88 (0.83–0.92)	5.00×10^{-8}
rs2043633	16	5759275	6	Yes	0.998	meta $P < 1 \times 10^{-4}$	0.88 (0.79–0.97)	.005	5	0.87 (0.82–0.92)	6.02×10^{-9}
rs3923500	13	85657509	7	Yes	1	meta $P < 1 \times 10^{-4}$	0.94 (0.80–1.07)	.336	5	1.13 (1.07–1.19)	5.96×10^{-5}
rs2731672	5	1.77E+08	8	Yes	0.999	meta $P < 1 \times 10^{-4}$	0.91 (0.81–1.02)	.082	5	0.88 (0.82–0.93)	1.07×10^{-6}
rs2701108	12	1.13E+08	9	Yes	0.993	meta $P < 1 \times 10^{-4}$	0.89 (0.80–0.98)	.011	5	0.88 (0.83–0.93)	1.39×10^{-7}
rs3734960	7	1.54E+08	10	Yes	0.999	meta $P < 1 \times 10^{-4}$	0.99(0.89–1.09)	.814	5	1.11 (1.05–1.16)	1.35×10^{-4}
rs6921589	6	25530348	11	Yes	1	meta $P < 1 \times 10^{-4}$	1.01 (0.88–1.14)	.880	5	1.15 (1.08–1.21)	6.48×10^{-5}
rs2218260	15	56001502	12	Yes	0.935	meta $P < 1 \times 10^{-4}$	1.05 (0.96–1.14)	.313	5	1.11 (1.06–1.16)	1.11×10^{-5}
rs4792891	17	41329294	13	Yes	0.995	meta $P < 1 \times 10^{-4}$	1.00 (0.91–1.09)	.971	5	0.91 (0.86–0.96)	1.44×10^{-4}

rs6480314	10	69637453	14	Yes	0.989	discovery $P < 1 \times 10^{-4}$, not on immunochip	1.03 (0.90–1.16)	.659	2	1.16 (1.08–1.24)	2.02×10^{-4}
rs9404438	6	1.04E+08	15	Yes	0.989	discovery $P < 1 \times 10^{-4}$, not on immunochip	0.99 (0.88–1.09)	.832	2	0.89 (0.82–0.96)	5.08×10^{-4}
rs7825744	8	72180729	16	Yes	1	meta $P < 1 \times 10^{-4}$	1.08 (0.93–1.23)	.316	5	1.18 (1.1–1.26)	2.43×10^{-5}
rs1473857	3	1.03E+08	17	Yes	1	meta $P < 1 \times 10^{-4}$	0.98 (0.87–1.10)	.788	5	1.11 (1.05–1.17)	4.43×10^{-4}
rs4674923	2	2.25E+08	18	Yes	0.999	discovery $P < 1 \times 10^{-4}$, not on immunochip	1.08 (0.98–1.17)	.141	2	1.08 (1.02–1.14)	1.24×10^{-2}
rs2319335	3	1.49E+08	19	Yes	0.998	discovery $P < 1 \times 10^{-4}$, not on immunochip	0.89 (0.78–1.00)	.036	2	1.07 (1.01–1.14)	4.02×10^{-2}
rs9074	20	44122072	20	Yes	1	meta $P < 1 \times 10^{-4}$	1.02 (0.92–1.12)	.676	5	0.91 (0.86–0.97)	6.90×10^{-4}
rs6032951	20	10717074	21	Yes	<90%[a]	meta $P < 1 \times 10^{-4}$	1.06 (0.95–1.18)	.295	5	1.13 (1.07–1.19)	3.16×10^{-5}
rs4742902	9	1.06E+08	22	Yes	1	meta $P < 1 \times 10^{-4}$	1.01 (0.91–1.11)	.815	5	0.91 (0.86–0.96)	5.12×10^{-4}
rs2425752	20	44135527	23	Yes	0.936	meta $P < 1 \times 10^{-4}$	0.97 (0.86–1.07)	.528	5	0.90 (0.85–0.95)	7.94×10^{-5}
rs1158793	4	96223364	24	Yes	0.962	discovery $P < 1 \times 10^{-4}$, not on immunochip	1.08 (0.97–1.19)	.171	2	0.87 (0.8–0.94)	3.43×10^{-5}
rs1357111	7	45651069	25	Yes	1	discovery $P < 1 \times 10^{-4,}$ not on immunochip	0.98 (0.82–1.14)	.796	2	0.86 (0.76–0.95)	1.65×10^{-3}
rs1333916	9	1.22E+08	26	Yes	1	meta $P < 1 \times 10^{-4}$	1.03 (0.93–1.13)	.582	5	0.92 (0.87–0.97)	1.03×10^{-3}
rs13087427	3	1.18E+08	27	Yes	0.995	meta $P < 1 \times 10^{-4}$	1.02 (0.88–1.16)	.776	5	0.88 (0.81–0.96)	7.29×10^{-4}

rs2854925	9	1.34E+08	28	Yes	0.988	discovery $P < 1 \times 10^{-4}$, not on immunochip	1.02 (0.92–1.11)	.759	2	1.10 (1.04–1.17)	1.65×10^{-3}
rs958889	11	1.28E+08	29	Yes	1	meta $P < 1 \times 10^{-4}$	0.99 (0.88–1.09)	.778	5	0.91 (0.85–0.96)	2.84×10^{-4}
rs12985909	19	18300383	30	Yes	0.999	meta $P < 1 \times 10^{-4}$	1.11 (1.02–1.20)	.027	5	1.11 (1.07–1.16)	4.20×10^{-6}
rs12156009	8	11322629	31	Yes	0.996	discovery $P < 1 \times 10^{-4}$, not on immunochip	0.97 (0.88–1.06)	.444	5	0.91 (0.87–0.96)	1.18×10^{-4}
rs4775330	15	59022174	32	Yes	0.998	meta $P < 1 \times 10^{-4}$	0.98 (0.88–1.07)	.641	5	1.08 (1.03–1.13)	1.24×10^{-3}
rs563198	9	77521795	33	Yes	0.999	meta $P < 1 \times 10^{-4}$	0.94 (0.85–1.04)	.245	5	0.90 (0.85–0.95)	5.38×10^{-5}
rs6926118	6	3320615	34	No	[a]	discovery $P < 1 \times 10^{-4}$, not on immunochip	0.98 (0.82–1.14)	.816	3	0.86 (0.76–0.95)	1.30×10^{-3}
rs7042370	9	12775073	35	Yes	0.999	discovery $P < 1 \times 10^{-4}$, not on immunochip	1.03 (0.93–1.12)	.619	2	0.90 (0.84–0.96)	4.84×10^{-4}
rs4858738	3	19826462	36	Yes	0.998	discovery $P < 1 \times 10^{-4}$, not on immunochip	1.04 (0.93–1.15)	.501	2	0.88 (0.81–0.95)	2.55×10^{-4}
rs1935671	20	8331096	37	Yes	0.863	meta $P < 1 \times 10^{-4}$	1.00 (0.89–1.10)	.935	4	1.10 (1.04–1.16)	8.66×10^{-4}
rs2902637	10	1.06E+08	38	No	[a]	meta $P < 1 \times 10^{-4}$	1.01 (0.90–1.11)	.905	5	1.10 (1.05–1.16)	5.71×10^{-4}
rs10903038	1	24363645	39	Yes	0.999	discovery $P < 1 \times 10^{-4}$, not on immunochip	0.98 (0.87–1.08)	.656	2	1.12 (1.05–1.18)	7.36×10^{-4}
rs851727	7	1.47E+08	40	Yes	1	meta $P < 1 \times 10^{-4}$	1.09 (0.87–1.30)	.444	5	0.85 (0.74–0.96)	2.76×10^{-3}
rs9379897	6	26709505	41	Yes	1	meta $P < 1 \times 10^{-4}$	0.86 (0.73–0.99)	.022	5	0.86 (0.79–0.92)	7.69×10^{-6}
rs9524596	13	94166840	42	Yes	0.999	meta $P < 1 \times 10^{-4}$	0.94 (0.85–1.03)	.156	5	0.91 (0.86–0.96)	5.69×10^{-5}

rs6903535	6	28525201	43	Yes	0.999	meta $P < 1 \times 10^{-4}$	0.98 (0.89–1.08)	.731	5	0.92 (0.87–0.97)	5.42×10^{-4}
rs6903130	6	32840188	44	No	NA	meta $P < 1 \times 10^{-4}$					
rs292808	1	33819233	45	No	NA	$P < 1 \times 10^{-4}$in sex-differentiated analysis of discovery					
rs10210285	2	1.43E+08	46	Yes	0.998	$P < 1 \times 10^{-4}$ in sex-differentiated analysis of discovery	1.06 (0.87–1.25)	.538	2	1.14 (1.02–1.26)	3.08×10^{-2}
rs7609738	3	1.83E+08	47	Yes	0.999	$P < 1 \times 10^{-4}$ in sex-differentiated analysis of discovery	1.06 (0.96–1.16)	.251	2	1.05 (0.99–1.11)	9.39×10^{-2}
rs7816766	8	4523552	48	Yes	0.991	$P < 1 \times 10^{-4}$in sex-differentiated analysis of discovery	0.88 (0.78–0.97)	.008	2	1.12 (1.06–1.18)	2.48×10^{-4}
rs719527	11	1.03E+08	49	Yes	0.998	$P < 1 \times 10^{-4}$in sex-differentiated analysis of discovery	0.99 (0.88–1.10)	.872	2	1.08 (1.01–1.15)	2.33×10^{-2}
rs2715425	15	97278619	50	Yes	0.998	*IGF1R* candidate variant	0.97 (0.86–1.09)	.647	1	0.97 (0.86–1.09)	.647
rs6898743	5	42638249	51	Yes	0.999	*GHR* candidate variant	1.03 (0.91–1.15)	.611	1	1.03 (0.91–1.15)	.611
rs1325190	1	1.98E+08	52	Yes	1	meta $P < 1 \times 10^{-4}$	1.05 (0.95–1.15)	.369	5	0.92 (0.87–0.97)	1.82×10^{-3}
rs7255	2	20742301	53	Yes	0.901	meta $P < 1 \times$	0.87 (0.78–0.96)	.003	5	0.87 (0.83–0.92)	1.09×10^{-8}

						10^{-4}					
rs13385191	2	20751746	54	Yes	0.999	meta $P < 1 \times 10^{-4}$	1.05 (0.94–1.15)	.375	5	1.11 (1.06–1.17)	1.06×10^{-4}
rs340620	2	20787591	55	Yes	0.982	meta $P < 1 \times 10^{-4}$	0.94 (0.84–1.03)	.175	5	0.90 (0.85–0.95)	1.52×10^{-5}
rs6727683	2	35429477	56	No	NA	meta $P < 1 \times 10^{-4}$					
rs12993283	2	35445909	57	Yes	0.999	meta $P < 1 \times 10^{-4}$	0.93 (0.84–1.02)	.121	5	0.89 (0.84–0.93)	2.15×10^{-7}
rs819848	3	1.57E+08	58	Yes	0.994	meta $P < 1 \times 10^{-4}$	1.02 (0.92–1.11)	.755	5	1.10 (1.05–1.15)	3.75×10^{-4}
rs9824398	3	1.88E+08	59	Yes	1	meta $P < 1 \times 10^{-4}$	1.06 (0.97–1.15)	.241	5	1.10 (1.05–1.15)	5.65×10^{-5}
rs9879899	3	1.88E+08	60	Yes	0.993	meta $P < 1 \times 10^{-4}$	0.95 (0.86–1.05)	.321	5	0.91 (0.86–0.96)	6.59×10^{-5}
rs13157599	5	1.23E+08	61	Yes	1	meta $P < 1 \times 10^{-4}$	0.92 (0.72–1.13)	.461	5	1.19 (1.08–1.29)	1.43×10^{-3}
rs35936561	7	1.56E+08	62	Yes	0.998	meta $P < 1 \times 10^{-4}$	0.98 (0.86–1.09)	.682	5	0.91 (0.86–0.96)	1.01×10^{-4}
rs7836059	8	11309574	63	No	NA	meta $P < 1 \times 10^{-4}$					
rs2898290	8	11471318	64	Yes	0.989	meta $P < 1 \times 10^{-4}$	0.92 (0.83–1.01)	.083	5	0.90 (0.86–0.95)	1.42×10^{-5}
rs12677326	8	11476634	65	No	NA	meta $P < 1 \times 10^{-4}$					
rs13267835	8	11542328	66	Yes	0.998	meta $P < 1 \times 10^{-4}$	0.97 (0.83–1.10)	.615	5	0.87 (0.80–0.94)	9.11×10^{-5}
rs13273672	8	11649790	67	Yes	0.999	meta $P < 1 \times 10^{-4}$	0.98 (0.89–1.08)	.739	5	1.09 (1.04–1.14)	6.90×10^{-4}
rs8180912	8	11677400	68	Yes	0.999	meta $P < 1 \times 10^{-4}$	1.00 (0.89–1.12)	.988	5	0.90 (0.84–0.96)	3.73×10^{-4}
rs7895043	10	71016742	69	Yes	0.999	meta $P < 1 \times 10^{-4}$	1.04 (0.95–1.13)	.428	5	1.09 (1.05–1.14)	1.52×10^{-4}
rs1265496	12	1.13E+08	70	No	NA	meta $P < 1 \times 10^{-4}$					

rs12903220	15	59040471	71	No	NA	meta $P < 1 \times 10^{-4}$					
rs7168393	15	95353461	72	Yes	0.999	meta $P < 1 \times 10^{-4}$	1.01 (0.85–1.17)	.866	5	1.14 (1.07–1.21)	1.96×10^{-4}
rs7173314	15	95353711	73	No	NA	meta $P < 1 \times 10^{-4}$					
rs2535483	15	95357916	74	No	NA	meta $P < 1 \times 10^{-4}$					
rs9941024	16	5734313	75	Yes	1	meta $P < 1 \times 10^{-4}$	0.90 (0.81–0.99)	.030	5	0.89 (0.84–0.94)	6.72×10^{-7}
rs7200175	16	5742063	76	No	NA	meta $P < 1 \times 10^{-4}$					
rs11866983	16	5743925	77	Yes	0.997	meta $P < 1 \times 10^{-4}$	1.09 (0.99–1.19)	.088	5	1.12 (1.07–1.17)	4.93×10^{-6}
rs9936833	16	84960619	78	Yes	0.997	meta $P < 1 \times 10^{-4}$	1.02 (0.91–1.14)	.669	5	1.15 (1.1–1.20)	4.68×10^{-8}
rs1532167	16	84961705	79	No	NA	meta $P < 1 \times 10^{-4}$					
rs7187365	16	85069416	80	No	NA	meta $P < 1 \times 10^{-4}$					
rs889592	16	85076757	81	Yes	1	meta $P < 1 \times 10^{-4}$	0.99 (0.87–1.11)	.921	5	0.90 (0.83–0.96)	3.00×10^{-4}
rs4792827	17	41487141	82	No	NA	meta $P < 1 \times 10^{-4}$					
rs6040146	20	10701237	83	No	NA	meta $P < 1 \times 10^{-4}$					

NOTE. Dutch Replication (Replication Phase 1) did not include the Dutch Extension at this stage (the Dutch Extension was just for the 7 SNPs taken through to Replication Phase 3, as outlined in Figure 1). rs41341748 was also typed in UK Replication Phase 3 (OR = 1.07; 95% CI: 0.70–1.43; $P_{meta} = .79$).

[a]SNPs in top 40 that could not be genotyped by Sequenom were genotyped by KASPar. KASPar call rates were all >95%. SNPs highlighted in light gray were excluded because of call rates <95%, SNPs highlighted in dark gray failed at the design stage of the iPLEX. SNPs were selected based on the following criteria: meta $P < 10^{-4}$ = $P_{association} < 10^{-4}$ in combined Discovery and Replication Phase 1 analysis, as described in Su et al[1] (n = 63); discovery $P < 10^{-4}$, not on immunochip = $P_{association} < 10^{-4}$ in Discovery Phase, but not included in the Immunochip content (n = 12); $P < 10^{-4}$ in sex-differentiated analysis of discovery =$P_{association} < 1 \times 10^{-4}$ in a sex-stratified analysis of the Discovery phase (n = 5); and candidate variant = candidate polymorphisms that had previously been reported to be associated with BE and were not well tagged by the Discovery Phase or Immunochip arrays.

Chr, chromosome.

Supplementary Table 2. Characteristics of Phase 3 Samples After Quality Control Exclusions

	UK Replication 3		Belgian Replication		Dutch Extension		BEACON/ BEAGESS	
	997 Cases	947 Control s	341 Cases	848 Controls	64 Cases	206 Controls	3295 Cases	3204 Controls
Sex								
Male	707	0	225	404	18	127	2489	2325
Female	274	947	74	444	2	79	806	879
Not stated	16	0	42	0	44		0	0
Age at diagnosis, y								
≤40	16				0			
41–50	73				1			
51–60	200				5			
61–70	397				7			
70+	286				7			
Not reported	25	947	341	848	44	206	3295	3204
Ethnicity								
White	934	947	341	848	64	206	3295	3204
Other	0	0	0	0	0	0	0	0
Not reported	63	0	0	0	0	0	0	0
Case only:								
Intestinal metaplasia	603	NA	341	NA	4	NA	3295	NA

Supplementary Table 3. Cohort Breakdown for the 7 Selected SNPs Taken Into Replication Phase 3

SNP	Chr	Position (build 37)	Minor/ major	BE meta	Meta I^2	n	UK Discovery	Dutch	UKREP1	Irish	UKREP2	UKREP3	Belgian	BEACON
rs3072	2	20878406	G/A	1.14<br>(1.09–1.18)<br>1.75×10^{-11}	0.42	8	1.23<br>(1.14–1.33<br>2.64×10^{-7}<br>0.41/0.36	1.20<br>(1.05–1.38<br>8.77×10^{-3}<br>0.41/0.36	1.06<br>(0.97–1.16)<br>2.22×10^{-1}<br>0.38/0.37	1.02<br>(0.81–1.30<br>8.47×10^{-1}<br>0.35/0.35	1.15<br>(1.04–1.28<br>4.79×10^{-3}<br>0.41/0.37	1.18<br>(1.04–1.34<br>1.18×10^{-2}<br>0.40/0.36	0.95<br>(0.79–1.14)<br>5.58×10^{-1}<br>0.36/0.38	1.11<br>(1.03–1.19)<br>6.64×10^{-3}
rs6751791	2	35581997	A/G	1.08<br>(1.04–1.12)<br>7.65×10^{-5}	0.60	8	1.15<br>(1.06–1.23<br>5.03×10^{-4}<br>0.49/0.48	1.10<br>(0.96–1.27<br>1.51×10^{-1}<br>0.50/0.47	1.18<br>(1.08–1.29)<br>3.05×10^{-4}<br>0.48/0.48	1.30<br>(1.04–1.63<br>2.22×10^{-2}<br>0.45/0.49	1.03<br>(0.93–1.13<br>5.97×10^{-1}<br>0.48/0.48	0.98<br>(0.83–1.16<br>8.04×10^{-1}<br>0.48/0.49	0.96<br>(0.80–1.14)<br>6.29×10^{-1}<br>0.48/0.49	1.00<br>(0.93–1.07)<br>8.92×10^{-1}
rs2731672	5	17684247<br>4	A/G	1.07<br>(1.03–1.12)<br>1.66×10^{-3}	0.62	8	1.18<br>(1.09–1.28<br>1.64×10^{-4}<br>0.27/0.24	1.10<br>(0.94–1.29<br>2.18×10^{-1}<br>0.27/0.25	1.15<br>(1.04–1.28)<br>8.03×10^{-3}<br>0.27/0.25	1.05<br>(0.82–1.35<br>6.82×10^{-1}<br>0.28/0.27	1.10<br>(0.99–1.23<br>8.51×10^{-2}<br>0.27/0.25	1.02<br>(0.85–1.24<br>8.13×10^{-1}<br>0.26/0.25	0.90<br>(0.73–1.10)<br>2.98×10^{-1}<br>0.25/0.27	0.95<br>(0.88–1.03)<br>2.09×10^{-1}
rs2701108	12	11467426<br>1	G/A	0.90<br>(0.86–0.93)<br>7.48×10^{-9}	0.14	8	0.88<br>(0.81–0.95<br>1.00×10^{-3}<br>0.38/0.41	0.91<br>(0.79–1.04<br>1.62×10^{-1}<br>0.37/0.39	0.86<br>(0.78–0.94)<br>9.76×10^{-4}<br>0.37/0.4	0.71<br>(0.57–0.89<br>2.86×10^{-2}<br>0.34/0.42	0.93<br>(0.84–1.03<br>1.46×10^{-1}<br>0.39/0.41	0.97<br>(0.86–1.10<br>6.69×10^{-1}<br>0.40/0.40	0.95<br>(0.80–1.14)<br>6.04×10^{-1}<br>0.39/0.41	0.91<br>(0.85–0.98)<br>1.43×10^{-2}
rs189247	15	97586630	A/G	1.10<br>(1.06–1.14)<br>3.55×10^{-7}	0.20	8	1.18<br>(1.09–1.27<br>5.67×10^{-5}<br>0.41/0.37	1.23<br>(1.06–1.41<br>5.00×10^{-3}<br>0.41/0.38	1.10<br>(1.00–1.21)<br>4.36×10^{-2}<br>0.40/0.38	1.03<br>(0.81–1.30<br>8.21×10^{-1}<br>0.41/0.40	1.11<br>(1.00–1.23<br>4.02×10^{-2}<br>0.39/0.37	1.07<br>(0.94–1.22<br>2.80×10^{-1}<br>0.40/0.38	1.00<br>(0.84–1.19)<br>9.89×10^{-1}<br>0.41/0.41	1.04<br>(0.97–1.12)<br>3.10×10^{-1}
rs2043633	16	5819274	C/A	0.92<br>(0.88–0.95)<br>2.25×10^{-6}	0.58	8	0.85<br>(0.79–0.92<br>6.04×10^{-5}<br>0.37/0.41	0.84<br>(0.74–0.97<br>1.36×10^{-2}<br>0.39/0.42	0.90<br>(0.82–0.98)<br>2.05×10^{-2}<br>0.38/0.40	0.87<br>(0.70–1.09<br>2.28×10^{-1}<br>0.35/0.39	0.88<br>(0.80–0.97<br>1.21×10^{-2}<br>0.38/0.41	1.03<br>(0.90–1.17<br>6.83×10^{-1}<br>0.39/0.38	0.84<br>(0.70–1.00)<br>5.59×10^{-2}<br>0.38/0.42	1.01<br>(0.94–1.09)<br>7.87×10^{-1}
rs12985909	19	18439383	G/A	1.10<br>(1.06–1.14)<br>3.28×10^{-7}	0.00	8	1.12<br>(1.04–1.21<br>2.94×10^{-3}<br>0.48/0.45	1.14<br>(0.99–1.30<br>6.06×10^{-2}<br>0.49/0.46	1.11<br>(1.02–1.22)<br>1.87×10^{-2}<br>0.47/0.44	1.09<br>(0.87–1.38<br>4.44×10^{-1}<br>0.46/0.44	1.11<br>(1.01–1.22<br>3.80×10^{-2}<br>0.47/0.45	1.03<br>(0.91–1.16<br>6.47×10^{-1}<br>0.46/0.46	1.14<br>(0.95–1.37)<br>1.51×10^{-1}<br>0.50/0.47	1.07<br>(1.00–1.15)<br>5.20×10^{-2}

NOTE. All results are presented with respect to the minor allele.
rs6751791 was not genotyped in BEACON; data presented are for a proxy SNP: rs7598399; pairwise $r^2 = 1$. rs189247 was not genotyped in BEACON, but was imputed from 4 genotyped SNPs (rs991757, rs2670927, rs2670930, and rs234540). Imputation accuracy using this strategy was 98.2%, confirmed by imputing samples for which genotypes from sequence data were available for all 5 SNPs and checking concordance of imputed and sequenced genotypes. Dutch cohort consists of Dutch Replication (Phase 1 replication) and Dutch Extension (Phase 3 replication). Columns for the BE meta and each sample set show (top to bottom) are OR, 95% CI, and P_{assoc}; minor allele frequency cases/controls.
n, number of studies; I^2, heterogeneity index I^2.

Supplementary Table 4. Sex Stratified/Heterogeneity Analyses Including BEACON Data

SNP	Chr	Minor/ major allele	Male, OR (95% CI) *P* value	Male studies, n	Female, OR (95% CI) *P* value	Female studies, n	Sex, differentiated *P* value	Sex, heterogeneity *P* value
rs3072	2	G/A	1.14 (1.09–1.19) 8.37×10^{-8}	7	1.08 (1.01-1.15) 1.57×10^{-2}	8	3.06×10^{-8}	.179
rs2701108	12	G/A	0.90 (0.85–0.94) 4.60×10^{-6}	7	0.88 (0.83-0.94) 4.77×10^{-5}	8	6.90×10^{-9}	.691

NOTE. Female analysis includes all cohorts (n = 8). Male analysis includes all except UK Replication Phase 3, as all controls were female (n = 7). Chr, chromosome.

Supplementary Table 5. Functional Annotation of SNPs in LD ($r^2 > 0.4$) With rs3072 Using Data From Haploreg, Phastcons, GERP, and RNA Seq Data Available From the ENCODE Project

rs number	Chr	Position (build 37)	r^2	D'	REF	ALT	EUR freq	Location	Refseq genes	GERP	Phast Cons	ENCODE RNA-seq RegTFBS -clustered	Promoter histone marks	Enhancer histone marks	DNAse	eQTL tissues	Motifs changed
rs9306894	2	20878105	0.97	0.99	A	G	0.36	Intergenic	GDF7(dist=6855), C2orf43(dist=6713)					GM12878	NHDF-neo		5 altered motifs
rs9306895	2	20878153	0.97	1	T	C	0.36	Intergenic	GDF7(dist=6903), C2orf43(dist=6665)					GM12878	NHDF-neo		HMG-IY,Lhx3,Pou3f3
**rs3072**	**2**	**20878406**	**1**	**1**	**T**	**C**	**0.36**	**Intergenic**	**GDF7(dist=7156), C2orf43(dist=6412)**					**GM12878**			**GATA,Gfi1**
rs7255	2	20878820	0.6	–0.95	T	C	0.53	Intergenic	GDF7(dist=7570), C2orf43(dist=5998)	2.28	Score 501; lod 145			GM12878			GZF1,Gm397,PLZF
rs10193919	2	20880833	0.92	1	C	T	0.35	Intergenic	GDF7(dist=9583), C2orf43(dist=3985)								6 altered motifs
rs2289081	2	20881840	0.87	0.95	G	C	0.36	Intergenic	GDF7(dist=10590), C2orf43(dist=2978)								4 altered motifs
rs13394027	2	20882056	0.5	0.97	G	A	0.23	Intergenic	GDF7(dist=10806), C2orf43(dist=2762)						30 cell types	4 eQTL tissues	Pbx3
rs10170771	2	20883216	0.47	0.89	T	C	0.25	Intergenic	GDF7(dist=11966), C2orf43(dist=1602)							Gibbs_FrontalCortex,Gibbs_Pons,Gibbs_TemporalCortex	4 altered motifs
rs12622106	2	20883561	0.47	0.89	C	T	0.25	Intergenic	GDF7(dist=12311), C2orf43(dist=12			JunD,c-Jun				Gibbs_FrontalCortex,Gibbs_Pons,Gi	

									57)							bbs_TemporalCortex	
rs10171934	2	20884546	0.48	0.92	A	C	0.25	Downstream	C2orf43			p300,FOXA1_ (C-20)		HepG2	HepG2		CEBPA
rs10182643	2	20884586	0.48	0.92	G	C	0.25	Downstream	C2orf43			SP1,p300, FOXA1_ (C-20)		HepG2	HepG2	Gibbs_FrontalCortex,Gibbs_Pons,Gibbs_TemporalCortex	NRSF,STAT
rs13385191	2	20888265	0.46	0.88	A	G	0.25	Intronic	C2orf43	2.86						Gibbs_FrontalCortex,Gibbs_Pons,Gibbs_TemporalCortex	Esr2
rs1437405	2	20929067	0.42	0.72	T	C	0.32	Intronic	C2orf43					HepG2, Huvec		Gibbs_FrontalCortex	22 altered motifs
rs2046325	2	20939706	0.41	0.71	T	C	0.32	Intronic	C2orf43								12 altered motifs

NOTE. Gene-based location, distance from Refseq genes, Phast cons, GERP, and SiPhy scores were obtained using annovar and hg19_refGene.txt and hg19_phastConsElements46way.txt and hg19_gerp++gt2.txt. All SNPs with blank GERP scores map to a location that score <2 and so is regarded as not being evolutionarily conserved/potentially functional. All SNPs with blank Phastcons scores represent SNPs that do not map to within conserved regions. None of the SNPs scored according to SiPhy database ljb2_siphy.txt. The Human Protein Atlas showed C2orf43to be expressed at moderate levels in normal squamous oesophageal epithelial cells and normal glandular stomach, but there was no expression data for the secreted protein GDF7.

Supplementary Table 6. Functional Annotation of SNPs in LD ($r^2 > 0.4$) With rs2701108 Using Data From Haploreg, Phastcons, GERP, SiPhy, and RNA seq Data Available From the ENCODE Project

rs no.	Chr	position (build 37)	r2	D'	REF	ALT	EUR freq	Location	Refseq genes	GERP	Phast Cons	ENCODE RNA-seq RegTFBS-clustered	Promoter histone marks	Enhancer histone marks	DNAse	eQTL tissues	Motifs changed
**rs1920562**	**12**	**114660658**	**0.62**	**0.81**	**T**	**C**	**0.35**	**Intergenic**	**RBM19(dist=256482), TBX5(dist=131077)**		**Score 556; lod 243**			**H1, NHLF**			**5 altered motifs**
rs12828548	12	114661066	0.62	0.81	C	G	0.35	Intergenic	RBM19(dist=256890), TBX5(dist=130669)	2.1				H1			Foxj1,Rad21,Zfp105
rs11066998	12	114661789	0.6	0.8	A	T	0.35	Intergenic	RBM19(dist=257613), TBX5(dist=129946)								Bach2
rs2555009	12	114666099	0.51	0.98	A	G	0.52	Intergenic	RBM19(dist=261923), TBX5(dist=125636)					HepG2			
rs11067002	12	114667046	0.83	0.96	T	A	0.34	Intergenic	RBM19(dist=262870), TBX5(dist=124689)								CIZ, Evi-1
rs2555019	12	114668618	0.52	0.99	T	C	0.52	Intergenic	RBM19(dist=264442), TBX5(dist=123117)	2.13							4 altered motifs
rs1920568	12	114669732	0.58	0.99	G	C	0.49	Intergenic	RBM19(dist=265556), TBX5(dist=122003)	2.29							EWSR1FLI1, Ets,STAT
rs2555016	12	114670663	0.52	0.99	T	G	0.52	Intergenic	RBM19(dist=266487),								BRCA1, Evi-

									TBX5(dist=121072)								1,PEBP
rs2701109	12	114671825	0.52	0.99	T	A	0.52	Intergenic	RBM19(dist=267649), TBX5(dist=119910)								5 altered motifs
rs10850292	12	114672508	0.47	0.95	C	A	0.23	Intergenic	RBM19(dist=268332), TBX5(dist=119227)								7 altered motifs
rs1247943	12	114673421	0.53	0.99	G	A	0.52	Intergenic	RBM19(dist=269245), TBX5(dist=118314)								RFX5
rs1247942	12	114673723	0.96	0.99	G	C	0.37	Intergenic	RBM19(dist=269547), TBX5(dist=118012)								8 altered motifs
rs2555015	12	114673774	0.64	0.99	T	C	0.47	Intergenic	RBM19(dist=269598), TBX5(dist=117961)								Nkx2,Pbx3
**rs2701108**	**12**	**114674261**	**1**	**1**	**T**	**C**	**0.36**	**Intergenic**	**RBM19(dist=270085), TBX5(dist=117474)**								**4 altered motifs**
rs1270886	12	114676470	0.54	-0.85	C	T	0.57	Intergenic	RBM19 (dist=272294), TBX5(dist=115265)					K562			NRSF
rs1265496	12	114676983	0.55	0.85	C	T	0.43	Intergenic	RBM19(dist=272807), TBX5(dist=114752)								Pax-4
rs2555014	12	114677491	0.55	0.85	G	T	0.43	Intergenic	RBM19(dist=273315), TBX5(dist=114244)								Hoxb6,NRSF,Pdx1

rs2555013	12	114678318	0.56	0.99	T	C	0.5	Intergenic	RBM19(dist=274142), TBX5(dist=113417)								4 altered motifs
rs7980132	12	114678673	0.41	1	A	G	0.19	Intergenic	RBM19(dist=274497), TBX5(dist=113062)								
rs2555012	12	114678725	0.52	0.84	C	T	0.44	Intergenic	RBM19(dist=274549), TBX5(dist=113010)						Osteobl		GR,RXRA
rs2252414	12	114679137	0.52	0.84	G	A	0.44	Intergenic	RBM19(dist=274961), TBX5(dist=112598)								12 altered motifs
rs1950090	12	114680189	0.42	1	A	G	0.58	Intergenic	RBM19(dist=276013), TBX5(dist=111546)			USF-1,BCL3		K562	HFF-Myc,NT2-D1		10 altered motifs
rs11067013	12	114681027	0.41	1	C	T	0.19	Intergenic	RBM19(dist=276851), TBX5(dist=110708)								4 altered motifs
rs1270885	12	114681552	0.51	0.84	A	G	0.44	Intergenic	RBM19(dist=277376), TBX5(dist=110183)								CDP,Irx,RXRA
rs1247940	12	114682651	0.52	0.84	T	C	0.44	Intergenic	RBM19(dist=278475), TBX5(dist=109084)								Pax-5
rs34388546	12	114683214	0.41	1	C	T	0.19	Intergenic	RBM19(dist=279038), TBX5(dist=108521)					H1			Irx
rs2252923	12	114683320	0.48	0.79	A	G	0.42	Intergenic	RBM19(dist=279144),					H1			HNF4,Pax-2,RXRA

									TBX5(dist=108415)								
rs2252924	12	114683323	0.51	0.83	C	A	0.43	Intergenic	RBM19(dist=279147), TBX5(dist=108412)					H1			Pax-2
rs1247938	12	114683568	0.52	0.83	G	A	0.44	Intergenic	RBM19(dist=279392), TBX5(dist=108167)			CTCF,CTCF_(SC-5916),Rad21			12 cell types		NRSF,Spz1
rs1269789	12	114684542	0.52	0.83	T	C	0.44	Intergenic	RBM19(dist=280366), TBX5(dist=107193)								Irx
rs2253207	12	114685437	0.51	0.83	T	C	0.44	Intergenic	RBM19(dist=281261), TBX5(dist=106298)								Nkx2
rs1270884	12	114685571	0.54	0.98	A	G	0.5	Intergenic	RBM19(dist=281395), TBX5(dist=106164)								SRF,TFIIA
rs4007267	12	114685668	0.45	0.75	A	T	NA	Intergenic	RBM19(dist=281492),TBX5(dist=106067)								Cdx,Dbx1,Dbx2,Evi-1,Foxa,Foxd3,Foxo,Foxp1,HDAC2,HMG-IY,HNF1,Hoxd8,Ncx,Nkx6-1,PLZF,Pax-4,Pou2f2,Sox,TATA,Zfp105,p300

rs2555004	12	114686645	0.58	0.98	G	A	0.49	Intergenic	RBM19(dist=282469), TBX5(dist=105090)			NRSF					4 altered motifs
rs1247928	12	114686840	0.52	0.83	T	C	0.44	Intergenic	RBM19(dist=282664), TBX5(dist=104895)								YY1,Zfp691
rs1247927	12	114687056	0.52	0.83	T	C	0.44	Intergenic	RBM19(dist=282880), TBX5(dist=104679)								5 altered motifs
rs1247926	12	114687311	0.52	0.83	T	C	0.44	Intergenic	RBM19(dist=283135), TBX5(dist=104424)								Foxj1,GR, STAT
rs2701111	12	114664920	0.42	0.78	G	A	0.46	Intergenic	RBM19(dist=260744), TBX5(dist=126815)								5 altered motifs
rs11067004	12	114671418	0.47	0.95	A	G	0.23	Intergenic	RBM19(dist=267242), TBX5(dist=120317)								4 altered motifs
rs201604604	12	114676112	0.53	0.84	T	TAC	0.43	Intergenic	RBM19(dist=271936), TBX5(dist=115623)					K562			4 altered motifs
rs11382177	12	114676113	0.52	0.84	A	AC	0.44	Intergenic	RBM19(dist=271937), TBX5(dist=115622)					K562			5 altered motifs
rs201882298	12	114683319	0.51	0.83	T	TG	0.43	Intergenic	RBM19(dist=279143), TBX5(dist=108416)					H1			HNF4,Pax-2
rs200186585	12	114683322	0.49	0.79	AC	A	0.42	Intergenic	RBM19 (dist=279146),					H1			Pax-2

									TBX5(dist=108413)								
rs10706438	12	114685657	0.44	0.73	AT	A	0.41	Intergenic	RBM19(dist=281481), TBX5(dist=106078)								12 altered motifs
rs201288186	12	114685665	0.42	0.71	TTA	T	0.41	Intergenic	RBM19(dist=281489), TBX5(dist=106070)								20 altered motifs
rs35484355	12	114686244	0.51	0.83	GC	G	0.43	Intergenic	RBM19(dist=282068), TBX5(dist=105491)								LBP-1

NOTE. Gene-based location, distance from Refseq genes, Phast cons, GERP, and SiPhy scores were obtained using annovar and hg19_refGene.txt and hg19_phastConsElements46way.txt and hg19_gerp++gt2.txt. All SNPs with blank GERP scores map to a location that score <2 and so is regarded as not being evolutionarily conserved/potentially functional. All SNPs with blank Phastcons scores represent SNPs that do not map to within conserved regions. None of the SNPs scored according to SiPhy database ljb2_siphy.txt. The Human Protein Atlas showed TBX5 and RBM19 to be expressed at moderate levels in normal squamous oesophageal epithelial cells and normal glandular stomach cells.

NA, not available.

Supplementary Table 7. Pathways With Evidence of Enrichment of BE Risk Alleles From One or More Sources

Pathway (SNPs mapping to within 20 kb of a gene in the pathway were assigned to each pathway)	iGSEA4 GWAS FDR-corrected *P* value	SRT FDR-corrected *P* value	GenGen FDR-corrected *P* value
KEGG SYSTEMIC LUPUS ERYTHEMATOSUS	0	>.05	.102
REACTOME MEIOTIC RECOMBINATION	0	>.05	.223
REACTOME PACKAGING OF TELOMERE ENDS	0	>.05	>.25
REACTOME RNA POL I PROMOTER OPENING	0	0	>.25
REACTOME DEPOSITION OF NEW CENPA CONTAINING NUCLEOSOMES AT THE CENTROMERE	0	.029	>.25
REACTOME MEIOTIC SYNAPSIS	0	>.05	>.25
REACTOME TELOMERE MAINTENANCE	0	>.05	>.25
REACTOME AMYLOIDS	0	>.05	>.25
REACTOME RNA POL I TRANSCRIPTION	0	>.05	>.25
REACTOME RNA_POL I RNA POL III AND MITOCHONDRIAL TRANSCRIPTION	0	>.05	>.25
REACTOME MEIOSIS	.001	>.05	.181
REACTOME BIOSYNTHESIS OF THE N GLYCAN PRECURSOR DOLICHOL LIPID LINKED OLIGOSACCHARIDE LLO AND TRANSFER TO A NASCENT PROTEIN	.001	.046	>.25
REACTOME TRANSCRIPTION	.002	>.05	>.25
REACTOME ANTIGEN PRESENTATION FOLDING ASSEMBLY AND PEPTIDE LOADING OF CLASS I MHC	.002	.029	>.25
REACTOME CHEMOKINE RECEPTORS BIND CHEMOKINES	.005	>.05	>.25
KEGG TYPE I DIABETES MELLITUS	.006	0	.165
PID RHOA REG PATHWAY	.006	>.05	>.25
REACTOME CHROMOSOME MAINTENANCE	.008	>.05	.19
KEGG ANTIGEN PROCESSING AND PRESENTATION	.008	0	.211
REACTOME ASPARAGINE N LINKED GLYCOSYLATION	.013	>.05	>.25
ST WNT CA2 CYCLIC GMP PATHWAY	.013	>.05	>.25
REACTOME RIG I MDA5 MEDIATED INDUCTION OF IFN ALPHA BETA PATHWAYS	.018	>.05	>.25
KEGG AUTOIMMUNE THYROID DISEASE	.026	0	.213
PID REELINPATHWAY	.028	>.05	>.25

REACTOME GLYCOLYSIS	.036	>.05	>.25
KEGG BASE EXCISION REPAIR	.046	>.05	>.25
PID HIF2PATHWAY	>.05	.029	.231
KEGG GRAFT VERSUS HOST DISEASE	>.05	.044	.236
KEGG CELL ADHESION MOLECULES CAMS	>.05	.046	.242
BIOCARTA IL5 PATHWAY	>.05	0	>.25
KEGG VIRAL MYOCARDITIS	>.05	0	>.25
REACTOME LYSOSOME VESICLE BIOGENESIS	>.05	0	>.25
KEGG ALLOGRAFT REJECTION	>.05	.029	>.25
PID CXCR4 PATHWAY	>.05	.029	>.25
PID MET PATHWAY	>.05	.029	>.25
REACTOME ABACAVIR TRANSPORT AND METABOLISM	>.05	.029	>.25
REACTOME ADAPTIVE IMMUNE SYSTEM	>.05	.029	>.25
REACTOME MHC CLASS II ANTIGEN PRESENTATION	>.05	.029	>.25
REACTOME NGF SIGNALLING VIA TRKA FROM THE PLASMA MEMBRANE	>.05	.029	>.25
REACTOME SIGNALLING BY NGF	>.05	.029	>.25
KEGG ARRHYTHMOGENIC RIGHT VENTRICULAR CARDIOMYOPATHY ARVC	>.05	.044	>.25
REACTOME CASPASE MEDIATED CLEAVAGE OF CYTOSKELETAL PROTEINS	>.05	.044	>.25
REACTOME CHOLESTEROL BIOSYNTHESIS	>.05	.044	>.25
REACTOME ER PHAGOSOME PATHWAY	>.05	.044	>.25
REACTOME SYNTHESIS OF SUBSTRATES IN N GLYCAN BIOSYTHESIS	>.05	.044	>.25
BIOCARTA ASBCELL PATHWAY	>.05	.046	>.25
KEGG ENDOCYTOSIS	>.05	.046	>.25
REACTOME CLASS I MHC MEDIATED ANTIGEN PROCESSING PRESENTATION	>..05	.046	>.25
REACTOME IMMUNE SYSTEM	>.05	.046	>.25
REACTOME INTERFERON ALPHA BETA SIGNALING	>.05	.046	>.25
REACTOME MEMBRANE TRAFFICKING	>.05	.046	>.25
REACTOME RECRUITMENT OF NUMA TO MITOTIC CENTROSOMES	>.05	.046	>.25
REACTOME RETROGRADE NEUROTROPHIN SIGNALING	>.05	.046	>.25

NOTE. Gengen did not identify any pathway as significantly enriched at FDR-corrected $P < .05$. The 3 pathways shown in *yellow* were found to be significantly enriched by both iGSEA4GWAS and SNP ratio test at FDR-corrected $P < .05$, with supportive evidence from GenGen (FDR $P < .25$). PLINK set based tests were run for these 3 pathways. Pathways shown in *gray* were no longer significant at FDR-corrected $P < .05$ after removal of HLA/major histocompatibility complex (MHC) genes. None of the pathways in *yellow* were significant at FDR-corrected $P < .05$ by any method after removal of HLA/MHC genes. Gene set KEGG TYPE I DIABETES MELLITUS: HLA-DRB4, HLA-DRB5, LTA, HLA-DOA, HLA-DOB, HLA-DRB3, CD80, CD86, CD28, TNF, ICA1, IFNG, LOC652614, PTPRN2, HLA-C, HLA-B, INS, IL1B, HLA-DMB, PTPRN, HLA-DMA, HLA-F, HLA-E, HLA-A, IL2, HLA-DPA1, IL12B, HLA-DPB1, GAD1, HLA-DQA1, HLA-G, HLA-DQA2, IL12A, CPE, HLA-DQB1, GAD2, IL1A, HSPD1, PRF1, FAS, FASLG, GZMB, HLA-DRB1, HLA-DRA. Gene set KEGG ANTIGEN PROCESSING AND PRESENTATION: HLA-DOA, HLA-DOB, KLRC3, KLRD1, KLRC1, KLRC2, RFXAP, RFX5, IFNA5, IFNA4, IFNA2, IFNA1, LGMN, PSME3, CTSS, HLA-C, HLA-B, HLA-DMB, HLA-DMA, HLA-A, HSPA1L, HSPA1B, HSPA2, KIR2DS5, HLA-G, KIR3DL1, KIR3DL2, HSPA1A, RFXANK, CREB1, IFNA17, HSPA5, CD74, HSPA4, IFNA21, CTSL1, IFNA6, IFI30, IFNA7, IFNA8, IFNA10, IFNA13, CIITA, IFNA14, IFNA16, KIR2DL5A, HSPA6, HLA-DRB4, CD4, KIR2DL1, HLA-DRB5, LTA, KIR3DL3, HLA-DRB3, TAP2, KIR2DS4, TAPBP, CD8A, CD8B, TAP1, KIR2DS3, HSPA8, KIR2DL4, CANX, KIR2DS1, KIR2DL2, KIR2DL3, KLRC4, LOC652614, NFYC, HSP90AA1, NFYA, NFYB, HLA-F CTSB, HLA-E, CALR, HLA-DPA1, HLA-DPB1, HLA-DQA1, PDIA3, HLA-DQA2, HLA-DQB1 PSME1, PSME2, HSP90AB1, B2M, HLA-DRB1, HLA-DRA. Gene set KEGG AUTOIMMUNE THYROID DISEASE: HLA-DOA, HLA-DOB, CD80, CD86, CD28, IFNA5, IFNA4, IFNA2, TSHR, TSHB, IFNA1, HLA-C, HLA-B, HLA-DMBHLA-DMA, HLA-A, HLA-G, TG, CGA, IFNA17, IFNA21, IFNA6, IFNA7, PRF1, IFNA8, IFNA10, IFNA13, IFNA14, GZMB, IFNA16, TPO, IL10, CTLA4, HLA-DRB4, HLA-DRB5, HLA-DRB3, LOC652614, HLA-F, HLA-E, IL2, CD40LG, HLA-DPA1, CD40, HLA-DPB1, HLA-DQA1, HLA-DQA2, HLA-DQB1, FAS, FASLG, IL4, IL5, HLA-DRB1, HLA-DRA.

SNP, SNP ratio test.

Supplementary Table 8. Polymorphisms Previously Reported to Be Associated With Risk of BE in Studies Published Before February 2014

Study	Phenotype	Gene	Variant	Chr	EA	Population	Published OR	Published *P* value	Discovery OR	Discovery *P*	Meta OR	*P* value
Ren,[2] 2014	BE vs GERD	CDX1	rs3776082	5	A	109 cases, 223 controls, ethnicity not stated (American)	4.02 (1.80–9.00)	<.01	0.99	.72	—	—
			rs717746	5	T		3.65 (1.73–7.69)	<.01	1.01	.73	—	—
			rs3776083 (proxy rs3776084 r^2 = 1, D' = 1)	5	A		0.28 (0.13–0.59)	<.01	1.09	.02	—	—
Izakovicova Holla,[3] 2013	BE vs healthy controls	IL-1 gene cluster	rs1143634[a]	2	T	60 cases, 165 controls Caucasian (Czech)	0.56 (0.33–0.93)	.016[b]	1.01	.89	1.00	.95
Menke,[4] 2012	BE vs healthy controls	Myo9B	rs2305764	19	G	254 cases, 198 controls Caucasian (Dutch)	2.96 (1.10–7.99)	.032[b]	—	—	—	—
Menke,[5] 2012	BE vs healthy controls	EGR	rs4444903 *Proxy: rs3796944* (r^2 = 0.965)	4	G	246 cases 198 controls Caucasian (Dutch)	3.0[c] (1.5–6.2)	<.05	1.07	.48	—	—
Menke,[6] 2012	BE vs healthy controls	TNFβ	rs909253 (proxy rs1041981, r^2 = 0.933)	6	A	257 cases 197 controls Caucasian (Dutch)	1.98[c] (1.03–3.81)	.04	1.12	.005	1.07	3.14×10^{-4}
Babar,[7] 2012	BE vs healthy controls	IL-18RAP	rs917997	2	C	213 cases 242 controls Caucasian (Irish)	0.63[c] (0.40–0.8)	2×10^{-4}	1.06	.233	—	—
		IL-18 promoter	rs1946518	2	C		1.26[c] (1.01–1.57)	.049	0.95	.196	0.96	.19
Orloff,[8]	BE/OAC	MSR1	rs41341748	8	T	176 cases, 200	OR not	.006	—	—	—	—

2011	vs healthy controls					controls Caucasian (US)	reported. Higher frequency of variant in cases than controls					
van de Winkel,[9] 2011	BE vs healthy controls	PXR	rs3814055	3	T	249 cases	1.44 (0.99–2.10)	<.05	0.924	.38875	0.99	.715
			rs6785049 *Proxy rs6784598 (r^2 = 1)*	3	G	201 controls Caucasian (Netherlands)	1.36 (1.03–1.79)	<.05	1.12	.201	—	—
McElholm,[10] 2010	BE vs healthy controls	IGF1	rs6214	12	A	224 cases 260 controls Caucasian (Irish)	0.43[c] (0.24–0.75)	.005	1.02	.802	—	—
		GHR	rs6898743	5	C		Not provided	.018[b]	—	—	—	—
		IGF1R	rs2715425	15	T		Not provided	.013[b]	—	—	—	—
		IGF1R	rs4966044	15	A		Not provided	.011[b]	—	—	—	—
Bradbury,[11] 2009	BE vs healthy controls	MMP1	rs1799750	11	G	99 cases 455 controls Canadian, ethnicity not stated	1.38 (1.02–1.87)	.04	—		—	—
		MMP12	rs2276109 *Proxy rs17368659* r^2=1	11	G		1.66 (1.00–2.77)	.05	0.92	.5416	—	—
Macdonald,[12] 2009	BE vs healthy controls, stratified by BMI <30 vs BMI >30	IGF1R	rs2229765	15	A	125 cases 95 controls Ethnicity not stated (Canadian)	4.81[c] (1.09–21.15)	<.05	0.98	0.546	—	—
Moons,[13]	BE vs	IL-12B	rs3212227	5	C	255 cases, 247	1.8[c]	.007	0.91	.428	0.99	.715

2008	reflux esophagitis		*Proxy rs3213094* r^2 = 1			controls Caucasian (Dutch)	(1.2–2.7)					
di Martino,[14] 2007	BE vs healthy controls	NQO1	rs1800566	16	T	200 cases, 94 controls, Ethnicity not stated	0.22[c] (0.07–0.76)	.01	0.95	.637	—	—
Kala,[15] 2007	BE vs healthy controls	GSTP1	rs1138272	11	B	22 cases, 173 controls Caucasian (Czech)	2.56[a] (1.30–5.05)	<.05	0.78	.111	—	—
Casson,[16] 2005	BE vs healthy controls	CCND1	rs9344<br><br>*Proxy rs649392*<br><br>(r^2 = 0.736)	11	A	125 cases 95 controls Ethnicity not stated (Canadian)	3.69[c] (1.46–9.29)	<.05	1.08	.379	—	—
Casson,[17] 2005	BE vs healthy controls	XRCC1	rs25487<br><br>*Proxy rs1799778*<br><br>(r^2 = 0.966)	9	A	125 cases 95 controls Ethnicity not stated (Canadian)	0.38[c] (0.12–0.64)	<.05	1.04	.699	—	—
Gough,[18] 2005	BE vs esophagitis /OAC cases without evidence of BE	IL-10	rs1800896	1	2	153 cases 303 controls ethnicity not stated (British)	1.84[c] (1.04–3.28)	.035	0.99	.725	1.03	.37
van Lieshout,[19] 1999	BE vs healthy controls	GTSP1	Rs1695[d]	11	G	98 cases, 247 controls Caucasian (Dutch)	3.28 (1.95–5.51)	<.0001	1.02	.56	—	—

BMI, body mass index; Chr, chromosome; EA, effect allele; IL, interleukin.

[a]rs1143634 was also examined in combination with 3 other variants in the interleukin-1 gene cluster in a haplotype analysis with significant findings. Only rs1143634 is genotyped on our discovery arrays so it was not possible to repeat the haplotype analysis.

[b]No correction made for multiple comparisons; not significant if corrected via Bonferroni method.

[c]Adjusted.
[d]This SNP was also examined in a meta-analysis by Bull et al, which includes the van Lieshout study and 3 negative findings; meta OR = 1.5 (95% CI: 1.16–1.95).

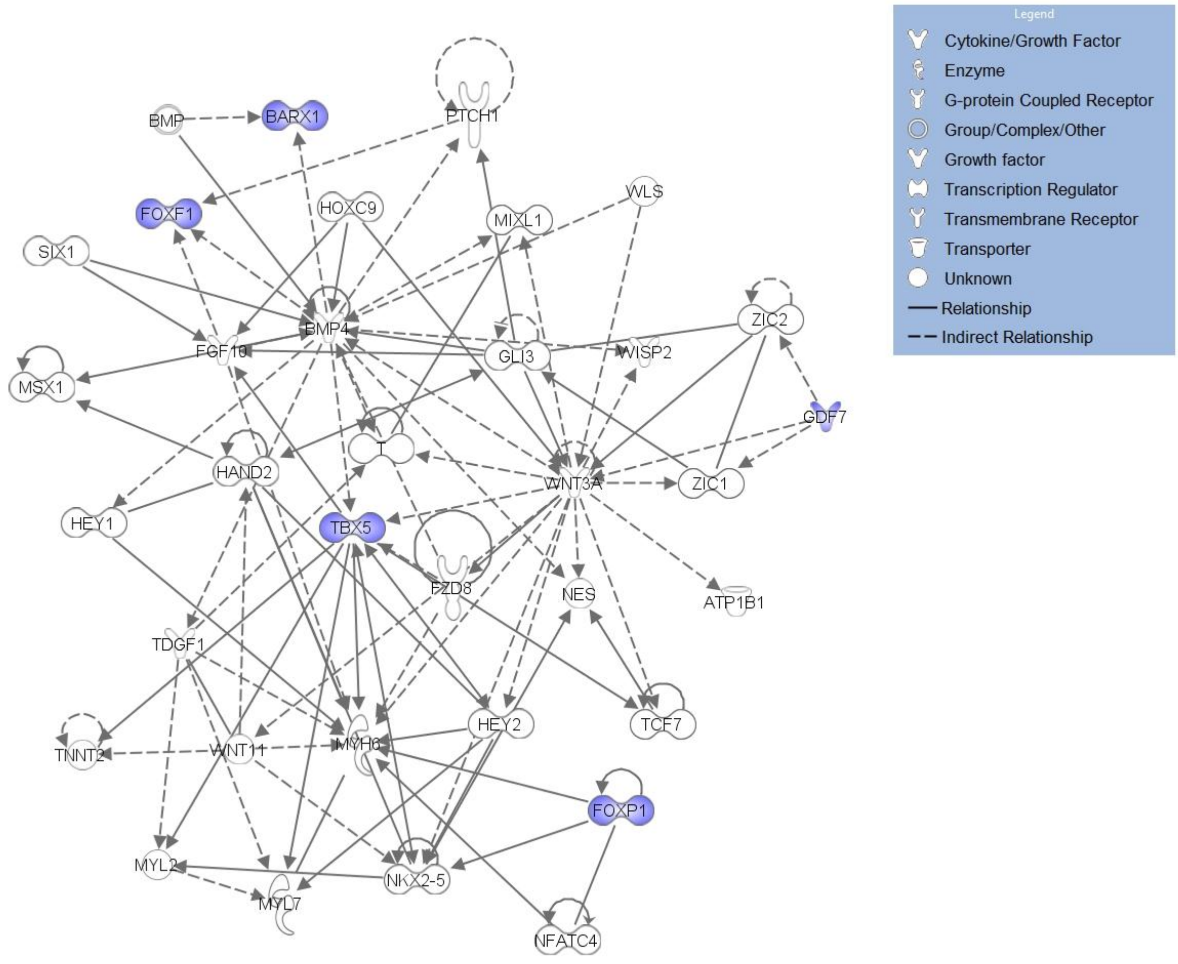

© 2000-2014 Ingenuity Systems, Inc. All rights reserved.

Supplementary Figure 1. Ingenuity Pathway Analysis network analysis of genes implicated by genome wide significant associations in BE case control analyses. Genes implicated by the SNPs shown to be associated with BE risk at genome-wide significance are highlighted in *blue*. A core analysis was run on these genes with confidence settings “experimentally observed only”; all other settings were default.

Symbol	Entrez gene name
ATP1B1	ATPase, Na+/K+ transporting, beta 1 polypeptide
BARX1	BARX homeobox 1
BMP	—
BMP4	bone morphogenetic protein 4
FGF10	fibroblast growth factor 10
FOXF1	forkhead box F1
FOXP1	forkhead box P1
FZD8	frizzled family receptor 8
GDF7	growth differentiation factor 7
GLI3	GLI family zinc finger 3
HAND2	heart and neural crest derivatives expressed 2
HEY1	hairy/enhancer-of-split related with YRPW motif 1
HEY2	hairy/enhancer-of-split related with YRPW motif 2
HOXC9	homeobox C9
MIXL1	Mix paired-like homeobox
MSX1	msh homeobox 1
MYH6	myosin, heavy chain 6, cardiac muscle, alpha
MYL2	myosin, light chain 2, regulatory, cardiac, slow
MYL7	myosin, light chain 7, regulatory
NES	nestin
NFATC4	nuclear factor of activated T-cells, cytoplasmic, calcineurin-dependent 4
NKX2-5	NK2 homeobox 5
PTCH1	patched 1
SIX1	SIX homeobox 1
T	T, brachyury homolog (mouse)
TBX5	T-box 5
TCF7	transcription factor 7 (T-cell specific, HMG-box)
TDGF1	teratocarcinoma-derived growth factor 1
TNNT2	troponin T type 2 (cardiac)
WISP2	WNT1 inducible signaling pathway protein 2
WLS	wntless homolog (Drosophila)
WNT11	wingless-type MMTV integration site family, member 11
WNT3A	wingless-type MMTV integration site family, member 3A
ZIC1	Zic family member 1
ZIC2	Zic family member 2

A:

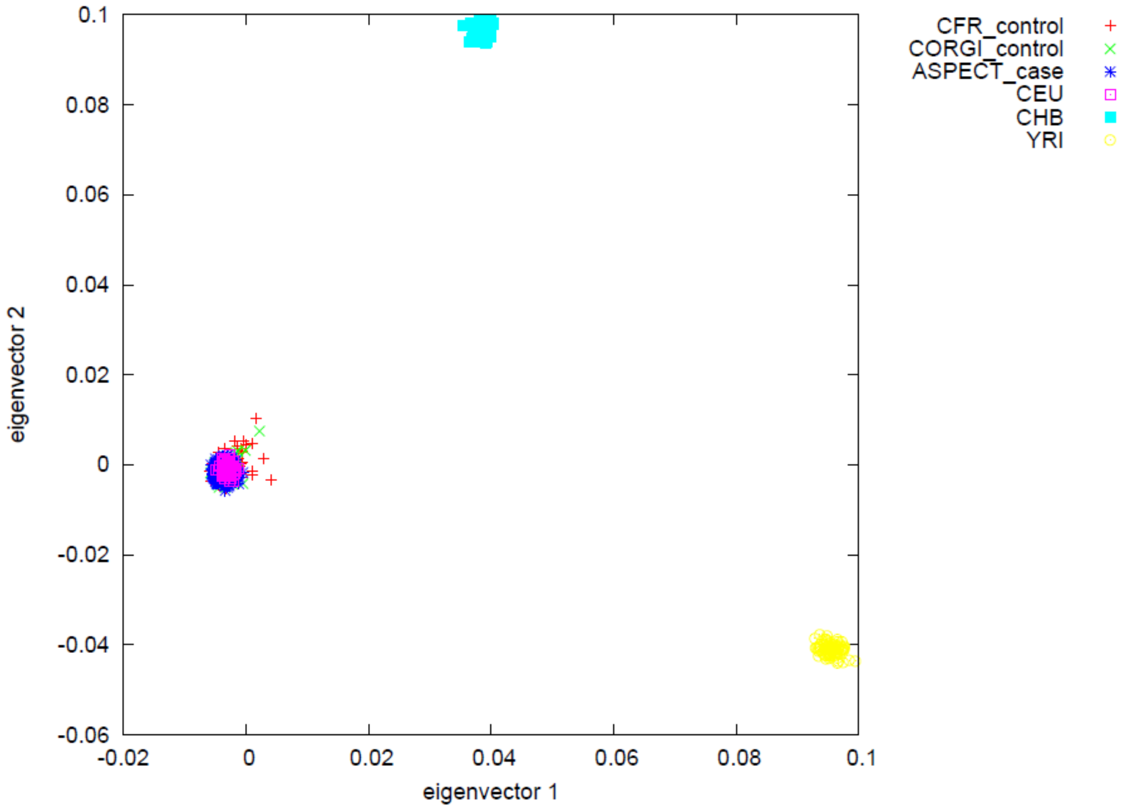
0.1
0.08
0.06
0.04
0.02
0
-0.02
-0.04
-0.06
-0.02
0
0.02
0.04
0.06
0.08
0.1
eigenvector 2
eigenvector 1
CFR_control
CORGI_control
ASPECT_case
CEU
CHB
YRI

B:

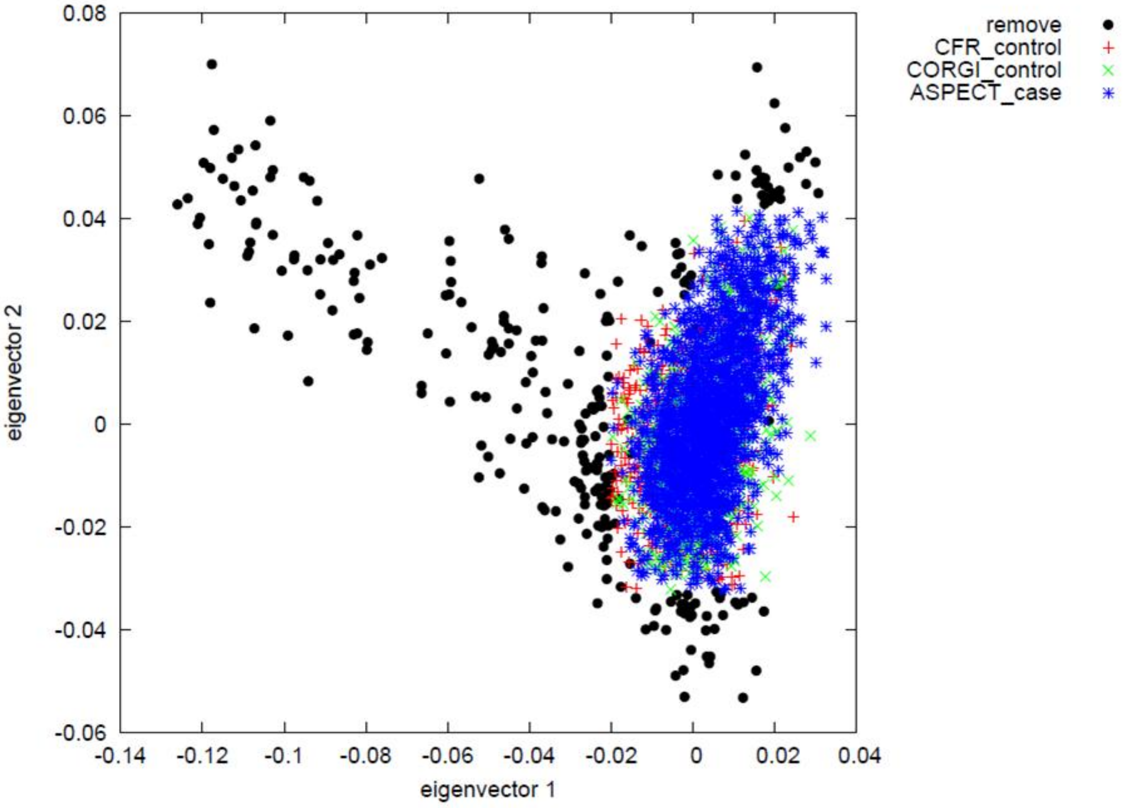
0.08
0.06
0.04
0.02
0
-0.02
-0.04
-0.06
-0.14
-0.12
-0.1
-0.08
-0.06
-0.04
-0.02
0
0.02
0.04
eigenvector 2
eigenvector 1
remove
CFR_control
CORGI_control
ASPECT_case

Supplementary Figure 2. (*A*) Principal component analysis (PCA) of AspECT cases (n = 1852), controls from the Colon Cancer Family Registry 1 (CFR1) and Colorectal Tumour Gene Identification (CoRGI) studies (n = 1898), and HapMap samples. (*B*) PCA of cases and controls only showing samples that were excluded from the analyses (n = 320) based on plotting PCs1–4 (PCs3 and 4 not shown). PC 1 was statistically significantly different in cases and controls and so was adjusted for in all analyses, no other PCs were significantly associated with case control status. Genomic inflation (λ = 1.068).

Ethical Considerations

The ethics of the project were reviewed by the East London and the City Research Ethics Committee (04/Q0603/1). All UK studies were performed with national ethics committee approval (MREC numbers: AspECT 04/Q0603/1; ChOPIN/IPOD 06/Q1603/07; HANDEL 09/H0505/23; CORGI 06/Q1702/99; and GLACIER 06/Q1702/64). The Irish samples were collected with approval from the Research Ethics Committee Board of St James's Hospital. The Dutch and Belgian replication samples were collected with approval from the ethics committee or Institutional Review Board of all participating institutions. The BEACON project was approved by the ethics boards of each participating institution. All study participants provided written informed consent.

Replication and Validation of Single Nucleotide Polymorphisms From BEACON/BEAGESS Meta-analysis

All subjects were self-described as white and had previously been analyzed in relation to HapMap samples to remove any non-Caucasian samples. Principal component analysis (PCA) showed that most of these cases and controls were genetically well matched and few outliers were detected following plotting of the first 10 PCs against one another (Supplementary Figure 2*B*). There were 1741 cases and 1642 controls included in the analysis. PC1 was significantly associated with case control status and so was included as a covariate in the association testing in the Discovery Phase. λ_{GC} was 1.077 before including the first PC and 1.068 after adjusting for this, suggesting that population structure was not a major confounder in our discovery phase. PCs 2–10 were not significantly associated with case-control status and the inclusion of these PCs had little or no effect on λ_{GC}. To deal with inter-study differences in array content, SNP genotypes were phased using SHAPEIT[28] and imputed using IMPUTE2[29] using recommended software options. The September 2013

release of the 1000 genomes project was used as a reference panel. SNPs with IMPUTE2 info scores of <0.95 or showing departures from Hardy-Weinberg equilibrium ($P < 10^{-6}$) were excluded.

In Silico Fine Mapping and Annotation of 1 Mb Regions Surrounding Lead Genotyped Single Nucleotide Polymorphisms

IMPUTE2 was used to impute 1 Mb surrounding the lead genotyped SNPs on chromosome 2p24.1 and chromosome 12q24.21. HapMap3 release 2 and the 2009 release of the 1000 genomes project were used as reference panels and recommended software parameters were applied. Hit plots of the two regions were created using LocusZoom,[20] LD information used was from the 2009 release of the 1000 genomes project, CEU samples. We tested SNPs using eQTL browsers, HaploRegv2,[21] Regulome DB,[22] SCAN, and Genevar.[23] For Genevar, all available databases were interrogated. Gene-based location, distance from Refseq genes, Phast cons, GERP, and SiPhy scores for each SNP were obtained using annovar[24] and hg19_refGene.txt and hg19_phastConsElements46way.txt and hg19_gerp++gt2.txt. Phast cons[25] is a phylogenetic hidden Markov model based program that uses multiway alignments to identify evolutionarily conserved regions of the genome, which better fit the conserved model than the nonconserved model. GERP[26] scores are measures of the rate of evolutionary constraint generated by quantifying base substitution deficits in multiway alignments, SiPhy[27] scores genomic regions based on the patterns of base substitutions rather than the rate.

Pathway/Gene Set Enrichment Analysis

We applied 3 pathway analysis methods to our Discovery dataset. Two of the methods, Gengen[28] and SNP ratio test[29] use SNP genotype data for phenotype-based permutation (1000 trials), while iGSEA4GWAS[30] is based on SNP *P* value permutation. SNPs mapping to

within 20 kb (upstream and downstream) of genes in 1320 canonical pathway gene sets from MSigDB v4.0 were used.

For iGSEA4GWAS, we input SNP identifiers, BE association *P* values and the 1320 gene sets to the website. Pathways with false discovery rate (FDR)–corrected $P < .05$ were selected as significant.

For Gengen, we performed 1000 permutations of case and control labels among the 7024 Discovery phase samples (1852 cases and 5172 controls), each time calculating the association *P* values using SNPTEST. Three perl scripts from the Gengen package were used, Scan_region.pl for mapping SNPs to genes and Calculate_gsea.pl and Combine_gsea.pl for gene set enrichment analysis (GSEA). Pathways with FDR-corrected $P < .05$ were selected as significant and those with FDR-corrected $P < .25$ were considered suggestive of enrichment.

For the SNP ratio test, the number of significant SNPs ($P < .05$) in a particular gene set was divided by the number of SNPs in that gene set. To obtain the distribution and nominal *P* values, we used the phenotype-based permutation *P* values calculated for Gengen. FDR-corrected $P < .05$ was chosen as the significance threshold for any gene set.

Pathways with an FDR-corrected *P* value <.05 according to iGSEA4GWAS and SNP ratio test and support from GenGen (FDR *P* value <.25) were tested in gene set–based tests in PLINK. After LD pruning using default settings, each set of SNPs was tested in a case-control analysis. All independent significant SNPs in a gene set were retained. The mean of these single SNP statistics was used as a set statistic. One thousand phenotype permutations were performed and the process of individual SNP testing repeated. An empirical *P* value for the gene set (EMP1) was calculated, representing the number of times the permuted set-statistic exceeds the real one.

Additionally, Ingenuity Pathway Analysis was performed for the genes implicated by the SNPs shown to be associated with BE risk at genome-wide significance. A core analysis was

run on a gene list, which consisted of *FOXF1*, *TBX5*, *GDF7*, *FOXP1*, and *BARX1*. Confidence settings were changed to “experimentally observed only,” all other settings were default.

Heritability Estimation by Genome-Wide Complex Trait Analysis

SNPs common to the Illumina 660W-Quad array and Illumina custom Human 1.2M-Duo array were analyzed in the Discovery Phase after applying strict genotyping quality control procedures. SNPs with a minor allele frequency of <1% and call rates of <98% had already been excluded for the GWAS analysis, as had individuals with a genotyping call rate of <99%. We additionally excluded all SNPs displaying departures from Hardy-Weinberg equilibrium at $P < 1 \times 10^{-4}$. Any related pairs of individuals were also removed before analysis. LDAK[31] was used to obtain kinship coefficients corrected for LD. Default settings were applied other than the additional use of the subset fixes to control for the fact that the cases and controls were genotyped separately. Restricted maximum likelihood analysis was performed using GCTA software.[32] Sex and 20 principal components, calculated using EIGENSTRAT/Smart PCA[33] were included as covariates, with BE case-control status as phenotype. A disease prevalence/lifetime risk of 1.6% was assumed.[34]

Expression Quantitative Trait Locus Analysis

We used the TCGA data portal (https://tcga-data.nci.nih.gov/tcga/dataAccessMatrix.htm?mode=ApplyFilter&diseaseType=ESCA) to download gene expression (RNASeq) data and SNP (Affymetrix 6.0) data from the esophageal adenocarcinoma sample set, and used the clinical data to select adenocarcinomas. We derived genomic segment copy number estimates from the SNP data and corrected transcript levels for these. We expressed SNP genotypes as $(N_{allele1})/(N_{allele1} + N_{allele2})$, to take

account of non-disomy. We tested for associations between genotype and transcript levels using linear regression and nonparametric trend tests. For specific genes of interest, associations were assessed using P = .05 as a threshold, whereas an FDR of q = .05 was used to assess SNPs as eQTLs genome-wide.

URLs

1000 Genomes: http://www.1000genomes.org/

PLINK: http://pngu.mgh.harvard.edu/~purcell/plink/

SNPTEST: https://mathgen.stats.ox.ac.uk/genetics_software/snptest/old/snptest.html

GTOOL: http://www.well.ox.ac.uk/~cfreeman/software/gwas/gtool.html

IMPUTE2: http://mathgen.stats.ox.ac.uk/impute/impute_v2.html

GWAMA: http://www.well.ox.ac.uk/gwama/index.shtml

LOCUS ZOOM: http://csg.sph.umich.edu/locuszoom/

ENCODE : http://genome.ucsc.edu/encode/

HAPLOREG: http://www.broadinstitute.org/mammals/haploreg/haploreg.php

SCAN: http://www.scandb.org/newinterface/about.html

Genevar: http://www.sanger.ac.uk/resources/software/genevar/

Regulome DB: http://www.regulomedb.org/

MSigDB v4.0: http://www.broadinstitute.org/gsea/msigdb/index.jsp

iGSEA4GWAS : http://gsea4gwas.psych.ac.cn/

GenGen: http://www.openbioinformatics.org/gengen/index.html

IPA: http://www.ingenuity.com/

Human Protein Atlas: http://www.proteinatlas.org/

Supplementary References

1. **Su Z, Gay** LJ, Strange A, et al. Common variants at the MHC locus and at chromosome 16q24.1 predispose to Barrett's esophagus. Nat Genet 2012;44:1131–1136.

2. Ren D, Zheng G, Bream S, et al. Single nucleotide polymorphisms of caudal type homeobox 1 and 2 are associated with Barrett's esophagus. Dig Dis Sci 2014;59:57–63.

3. Izakovicova Holla L, Borilova Linhartova P, Hrdlickova B, et al. Haplotypes of the IL-1 gene cluster are associated with gastroesophageal reflux disease and Barrett's esophagus. Hum Immunol 2013;74:1161–1169.

4. Menke V, Van Zoest KP, Moons LM, et al. Myo9B is associated with an increased risk of Barrett's esophagus and esophageal adenocarcinoma. Scand J Gastroenterol 2012;47:1422–1428.

5. Menke V, Pot RG, Moons LM, et al. Functional single-nucleotide polymorphism of epidermal growth factor is associated with the development of Barrett's esophagus and esophageal adenocarcinoma. J Hum Genet 2012;57:26–32.

6. Menke V, van Zoest KP, Moons LM, et al. NcoI TNF-beta gene polymorphism and TNF expression are associated with an increased risk of developing Barrett's esophagus and esophageal adenocarcinoma. Scand J Gastroenterol 2012;47:378–386.

7. Babar M, Ryan AW, Anderson LA, et al. Genes of the interleukin-18 pathway are associated with susceptibility to Barrett's esophagus and esophageal adenocarcinoma. Am J Gastroenterol 2012;107:1331–1341.

8. Orloff M, Peterson C, He X, et al. Germline mutations in MSR1, ASCC1, and CTHRC1 in patients with Barrett esophagus and esophageal adenocarcinoma. JAMA 2011;306:410–419.

9. van de Winkel A, Menke V, Capello A, et al. Expression, localization and polymorphisms of the nuclear receptor PXR in Barrett's esophagus and esophageal adenocarcinoma. BMC Gastroenterol 2011;11:108.

10. McElholm AR, McKnight AJ, Patterson CC, et al. A population-based study of IGF axis polymorphisms and the esophageal inflammation, metaplasia, adenocarcinoma sequence. Gastroenterology 2010;139:204–212 e3.

11. Bradbury PA, Zhai R, Hopkins J, et al. Matrix metalloproteinase 1, 3 and 12 polymorphisms and esophageal adenocarcinoma risk and prognosis. Carcinogenesis 2009;30:793–798.

12. MacDonald K, Porter GA, Guernsey DL, et al. A polymorphic variant of the insulin-like growth factor type I receptor gene modifies risk of obesity for esophageal adenocarcinoma. Cancer Epidemiol 2009;33:37–40.

13. Moons LM, Kusters JG, van Delft JH, et al. A pro-inflammatory genotype predisposes to Barrett's esophagus. Carcinogenesis 2008;29:926–931.

14. di Martino E, Hardie LJ, Wild CP, et al. The NAD(P)H:quinone oxidoreductase I C609T polymorphism modifies the risk of Barrett esophagus and esophageal adenocarcinoma. Genet Med 2007;9:341–347.

15. Kala Z, Dolina J, Marek F, et al. Polymorphisms of glutathione S-transferase M1, T1 and P1 in patients with reflux esophagitis and Barrett's esophagus. J Hum Genet 2007;52:527–534.

16. Casson AG, Zheng Z, Evans SC, et al. Cyclin D1 polymorphism (G870A) and risk for esophageal adenocarcinoma. Cancer 2005;104:730–739.

17. Casson AG, Zheng Z, Evans SC, et al. Polymorphisms in DNA repair genes in the molecular pathogenesis of esophageal (Barrett) adenocarcinoma. Carcinogenesis 2005;26:1536–1541.

18. Gough MD, Ackroyd R, Majeed AW, et al. Prediction of malignant potential in reflux disease: are cytokine polymorphisms important? Am J Gastroenterol 2005;100:1012–1018.

19. van Lieshout EM, Roelofs HM, Dekker S, et al. Polymorphic expression of the glutathione S-transferase P1 gene and its susceptibility to Barrett's esophagus and esophageal carcinoma. Cancer Res 1999;59:586–589.

20. Pruim RJ, Welch RP, Sanna S, et al. LocusZoom: regional visualization of genome-wide association scan results. Bioinformatics 2010;26:2336–2337.

21. Ward LD, Kellis M. HaploReg: a resource for exploring chromatin states, conservation, and regulatory motif alterations within sets of genetically linked variants. Nucleic Acids Res 2012;40:D930–D934.

22. Boyle AP, Hong EL, Hariharan M, et al. Annotation of functional variation in personal genomes using RegulomeDB. Genome Res 2012;22:1790–1797.

23. Yang TP, Beazley C, Montgomery SB, et al. Genevar: a database and Java application for the analysis and visualization of SNP-gene associations in eQTL studies. Bioinformatics 2010;26:2474–2476.

24. Wang K, Li M, Hakonarson H. ANNOVAR: functional annotation of genetic variants from high-throughput sequencing data. Nucleic Acids Res 2010;38:e164.

25. Siepel A, Bejerano G, Pedersen JS, et al. Evolutionarily conserved elements in vertebrate, insect, worm, and yeast genomes. Genome Res 2005;15:1034–10350.

26. Davydov EV, Goode DL, Sirota M, et al. Identifying a high fraction of the human genome to be under selective constraint using GERP++. PLoS Comput Biol 2010;6:e1001025.

27. Garber M, Guttman M, Clamp M, et al. Identifying novel constrained elements by exploiting biased substitution patterns. Bioinformatics 2009;25:i54–i62.

28. Wang K, Li M, Bucan M. Pathway-based approaches for analysis of genomewide association studies. Am J Hum Genet 2007;81:1278–1283.

29. O'Dushlaine C, Kenny E, Heron EA, et al. The SNP ratio test: pathway analysis of genome-wide association datasets. Bioinformatics 2009;25:2762–2763.

30. Zhang K, Cui S, Chang S, et al. i-GSEA4GWAS: a web server for identification of pathways/gene sets associated with traits by applying an improved gene set enrichment analysis to genome-wide association study. Nucleic Acids Res 2010;38:W90–W95.

31. Speed D, Hemani G, Johnson MR, et al. Improved heritability estimation from genome-wide SNPs. Am J Hum Genet 2012;91:1011–1021.

32. Lee SH, Wray NR, Goddard ME, et al. Estimating missing heritability for disease from genome-wide association studies. Am J Hum Genet 2011;88:294–305.

33. Price AL, Patterson NJ, Plenge RM, et al. Principal components analysis corrects for stratification in genome-wide association studies. Nat Genet 2006;38:904–909.

34. Ronkainen J, Aro P, Storskrubb T, et al. Prevalence of Barrett's esophagus in the general population: an endoscopic study. Gastroenterology 2005;129:1825–1831.

Author names in bold designate shared co-first authorship.
